# Hot Electrons in TiO_2_–Noble Metal Nano-Heterojunctions: Fundamental Science and Applications in Photocatalysis

**DOI:** 10.3390/nano11051249

**Published:** 2021-05-10

**Authors:** Ajay P. Manuel, Karthik Shankar

**Affiliations:** 1 Department of Electrical and Computer Engineering, University of Alberta, Edmonton, AB T6G 1H9, Canada; apmanuel@ualberta.ca; 2 Future Energy Systems Research Institute, University of Alberta, Edmonton, AB T6G 1K4, Canada

**Keywords:** solar energy conversion, charge transfer, oxide interfaces, optical resonances, Schottky barrier, nanoparticles, plasmon, TiO_2_, photoreduction, hot electron

## Abstract

Plasmonic photocatalysis enables innovation by harnessing photonic energy across a broad swathe of the solar spectrum to drive chemical reactions. This review provides a comprehensive summary of the latest developments and issues for advanced research in plasmonic hot electron driven photocatalytic technologies focusing on TiO_2_–noble metal nanoparticle heterojunctions. In-depth discussions on fundamental hot electron phenomena in plasmonic photocatalysis is the focal point of this review. We summarize hot electron dynamics, elaborate on techniques to probe and measure said phenomena, and provide perspective on potential applications—photocatalytic degradation of organic pollutants, CO_2_ photoreduction, and photoelectrochemical water splitting—that benefit from this technology. A contentious and hitherto unexplained phenomenon is the wavelength dependence of plasmonic photocatalysis. Many published reports on noble metal-metal oxide nanostructures show action spectra where quantum yields closely follow the absorption corresponding to higher energy interband transitions, while an equal number also show quantum efficiencies that follow the optical response corresponding to the localized surface plasmon resonance (LSPR). We have provided a working hypothesis for the first time to reconcile these contradictory results and explain why photocatalytic action in certain plasmonic systems is mediated by interband transitions and in others by hot electrons produced by the decay of particle plasmons.

## 1. Introduction

Solar energy is among the cleanest, and most abundant renewable energy sources available to the world. Our planet exploits solar energy routinely through photosynthesis—the process by which plants, algae, photosynthetic bacteria, and protists capture sunlight, water, and residual CO_2_ in our atmosphere as reactants for water-splitting chemistry:

6H_2_O + 6CO_2_ + Sunlight → C_6_H_12_O_6_ + 6O_2_

This process allows for the decomposition of water to molecular oxygen, and the transformation of CO_2_ to carbohydrates and other carbon-rich products integral to the sustainability of our planet’s biosphere [1].

Amidst the rapidly rising global energy demand (17.4 Terrawatts (TW) in 2015 and a 2.2% growth averaged in 2017, the fastest since 2013) [2] and environmental crises, the efficient utilization of solar energy in chemical transformations is extremely important for the modern energy industry. Global energy consumption is predicted to increase to about twice the current value in 2050 [3,4]. For decades, fossil fuels such as oil, coal, peat, and natural gas have served as conventional energy sources to meet the world’s energy demands and have provided for sustainable economic development. 140,000 TWh of energy per year is consumed by mankind with more than 80% accounted by fossil fuels. The dominance of fossil fuels in the global energy generation and distribution infrastructure is largely due to their availability, stability, and high energy density [5] but the proliferation of fossil fuel burning has led to a dramatic increase in atmospheric CO_2_ levels over the last century (up to 100 parts per million by volume) [6] with CO_2_ emissions widely considered as the major cause of global warming. Notwithstanding the increasing need to mitigate this global crisis, fossil fuels are also a limited energy source.

**Figure 1:**
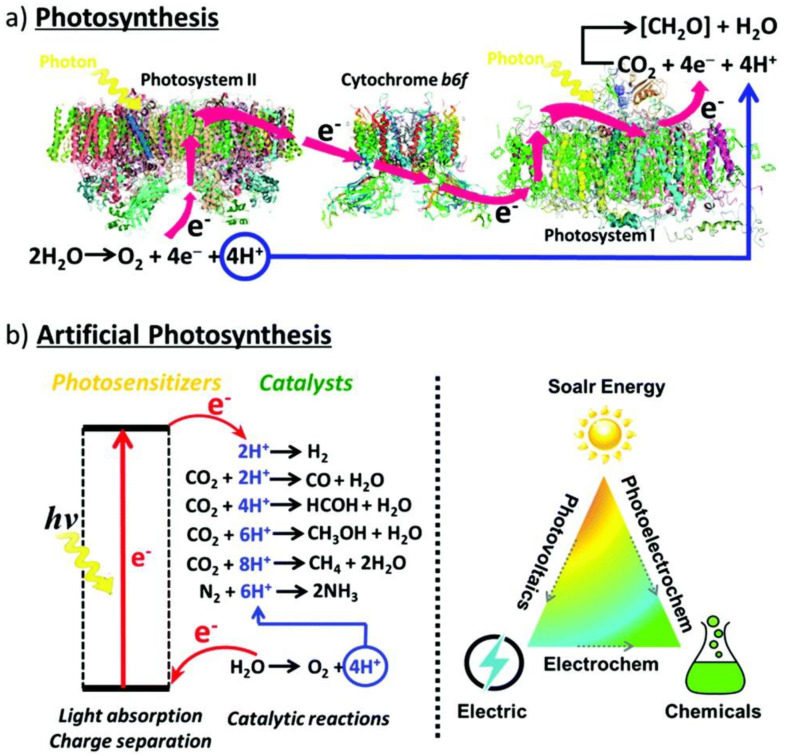
(**a**) Photosynthesis is enabled through the collaborative efforts of two photosynthetic complexes, PSI and PSII, where PSI serves as the reaction center and light harvesting complex and PSII is the site of water oxidation. Thus, H_2_O is oxidized in PSII into O_2_ releasing four protons and electrons, respectively, that are transferred via cytochrome *b6f,* an enzyme in plant chloroplasts, to PSI where they are consumed by CO_2_ reduction to produce carbohydrates. (**b**) Artificial photosynthetic systems for photocatalysis are being developed to mimic and provide for the very same conversion of solar energy through alternative energetic pathways and selectivity for fundamental and desirable chemical reactions, including water splitting, CO_2_ photoreduction, and the degradation of harmful organic pollutants. Reprinted with permission from Ref [10] with attribution and adherence to Creative Commons Attribution-NonCommercial 3.0 Unported Licence. Copyright Royal Society of Chemistry (2019).

To address these issues, considerable effort has been placed on the development of renewable, environment-friendly, artificial photosynthetic technologies to sustain modern technological civilization. The use of artificial photosynthetic technology is a means to not merely mimic photosynthesis but to improve our knowledge of the process and enhance it to our selective needs through artificial means (Figure 1). Solar irradiation on our planet in just one hour exceeds our annual energy consumption. By tapping into even 0.02% of the incoming solar energy, we could satisfy all our current energy needs [7,8]. In this objective, an assortment of technologies has been developed ranging from biological systems (algae), inorganic photocatalysts (transition metal oxides or semiconductors, particularly TiO_2_-based catalysts), organic photocatalysts (metal-organic complexes), biomimetic systems (enzyme-activated or dye-sensitized semiconductors), tandem cells, and z-schemes to name a few [6,9].

Over the recent decade, semiconductor photocatalysts have become highly popular as the key artificial photosynthetic technology and have set the basis for research in the field of photocatalysis. Researchers have developed many semiconductors as suitable candidates for photocatalysts including metal oxides, metal chalcogenides, metal nitrides, bismuth oxyhalides, carbon nitrides, and III-V compounds [11,12,13,14,15,16,17,18,19,20,21,22,23,24,25]. Semiconductor photocatalysts absorb photons to generate active electrons and holes that are then utilized to initiate chemical reduction and oxidation reactions [26,27,28]. A viable photocatalyst, in general, must allow for optimal light absorption (wide light-absorption range), efficient charge separation (good band energetics), charge migration for necessary chemical reactions (high carrier mobility and long carrier diffusion lengths), as well as strong catalytic activity, stability, and from a commercial viewpoint, high sustainability and low cost.

TiO_2_ has been the poster-child for semiconductor photocatalyst materials due to its relatively low cost, high availability, low toxicity, stability in both acidic and basic media, and resistance to photo-corrosion [29]. Yet, large-scale commercialization of semiconductor photocatalytic technology in the environmental and energy industries is still at its advent and remains to be fully exploited. This is because despite their obvious advantages, critical and debilitating material-sensitive limitations have surfaced over the years concerning semiconductor photocatalysts. Popular photocatalysts, such as TiO_2_ and SrTiO_3_, are relatively cheap, easy to process, and durable but are poor absorbers of visible light due to their wide bandgaps [30]. Contrastingly, narrow bandgap semiconductors, such as Si and Cu_2_O, lack long-term catalytic efficiency, and photo-corrode easily. Other semiconductors, such as Fe_2_O_3_, are inhibited by extremely low photocatalytic activity [30].

To overcome these limitations, plasmonic photocatalysts have emerged as a promising technology for harvesting and converting solar energy [31,32,33,34,35]. This is achieved by the generation and transfer of energetic charge carriers or “hot electrons” via resonant interaction of incident light with the collective and coherent motion of electrons in metal nanostructures to initiate, enhance, and promote photocatalytic activity. The exploitation of hot electrons produced by the localized surface plasmon resonance (LSPR) of noble metal nanoparticles in photocatalysis and photovoltaics has recently witnessed a surge of research interest [36,37,38,39,40,41,42]. The research interest is well-deserved since optimal exploitation of hot electrons holds out the promise of high performance, durable photocatalysts for water treatment, solar hydrogen generation from water splitting, and CO_2_ photoreduction. In spite of such intense research interest, many aspects related to the fundamental physics of hot electron generation and transfer from particle plasmons remain unclear. Our review is comprehensive and incorporates information from a broad cross-section of recent articles. Since the authors of this work have been researching the topic of noble metal nanoparticle, TiO_2_ heterojunction photocatalysts for water-splitting and CO_2_ photoreduction, we are well-placed to discuss the latest developments in this fast-changing field. One unique aspect of our review is that it has a self-contained section (Section 3) on “Probing Hot Electrons”, where we discuss in great detail the application of different spectroscopic techniques to characterize plasmonic hot electron photocatalysts and the interpretation of the characterization data thus obtained. We strongly believe this information will be valuable to both new researchers entering the field and even to experienced researchers who might have not considered a technique outside the suite of techniques they’re comfortable with.

### 1.1. What Are Hot Electrons and Why Are They Important?

Theoretical work on hot electrons began in the 1930s. Hot electrons can be generated by applying a strong electric field to a conductor. For metals, high electric fields may cause melting or result in extremely high joule heating. Therefore, most early hot electron work focused on understanding dielectric breakdown in insulators [43].

Hot electrons are essentially electrons that are not in thermal equilibrium with their immediate environment (generally, the atoms comprising a material) [43]. These electrons have a very high effective temperature (as high as several thousand Kelvin) compared to room temperature, due to their kinetic energy or resonant interaction/coupling with light. Hot electron lifetimes vary with respect to the relevant material structures. Hot electrons in bulk gold with an energy greater than 1 eV above the Fermi Energy (*E_F_*) have a lifetime smaller than 50 fs with the dominant relaxation mechanism being inelastic electron–electron interactions [44]. Meanwhile, due to reduced electron–electron interactions and confinement effects in small gold nanoparticles (Au NPs), hot electron lifetimes in Au NPs are typically an order of magnitude larger, in the range of 100–500 fs [45].

Both hot electrons and their counterparts in hot holes can be very effective in stimulating chemical and physical processes, being only limited by the rapid relaxation processes that accompany their emission. This very relaxation of the high energy carriers also helps stimulate heating of the solid structures involved. This fits the paradigm of photocatalysis as hot electrons can be utilized for various effects from local heating of particles and reactants to photochemistry, photodesorption, and controlled chemical reactions [46,47,48,49]. The discoveries of photochemical water splitting on TiO_2_ electrodes using ultraviolet light [50], surface-enhanced Raman spectroscopy (SERS) [51], and femtochemistry studies on single-crystal metal surfaces [52,53,54,55] served as foundational steps towards current interest in the utilization of hot electron induced chemical reactions on photoexcited metal surfaces, more precisely identified as plasmonic hot electron photocatalysis.

### 1.2. Plasmonic Hot Electron Photocatalysis Using TiO_2_–Noble Metal Nanostructures

Hot electron photocatalysts are typically composite systems that incorporate a semiconductor with a plasmonic noble metal nanostructure in a heterojunction. Plasmonic noble metal nanostructures have electron densities that can couple with wavelengths of electromagnetic radiation (in the visible spectral range) that are far larger than the nanostructure itself due to the dielectric-metal interface between the particles and the surrounding medium; contrastingly, in pure metals, there is a maximum limit on the magnitude of wavelengths (work-function dependent) that can effectively couple with the material sizes involved [56]. Plasmonic noble metal nanospheres have commonly been utilized as hot electron photocatalysts although recently diverse nanostructures, such as nanocubes, nanorods, nanoshells, gap plasmon structures, etc., have also been investigated [57,58,59,60].

**Figure 2:**
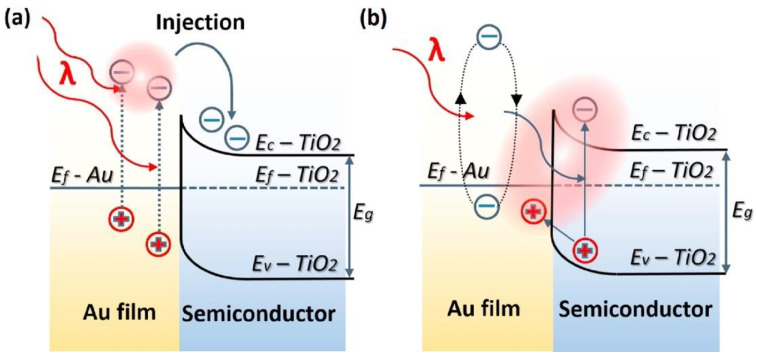
Energy band-diagram of Au and *n*-type TiO_2_ heterojunction showing LSPR-driven hot electron injection from Au into TiO_2_ by (**a**) over barrier thermionic emission and (**b**) tunneling mechanism. Note the bending of the conduction and valence bands of TiO_2_ at the contact interface of the two materials due to the equilibration of Fermi levels upon contact forming a Schottky barrier. *E*_F_, *E*_VB_, *E*_CB_, *ϕ*_B_, and *L* are the Fermi level, valence band level, conduction band level, Schottky barrier height, and the width of depletion layer, respectively. Reprinted with permission from Ref [70] Copyright Elsevier (2017).

Plasmon-enhanced photocatalysis capitalizes on the resonant interaction of light with the collective and coherent motion of electrons in the noble metal nanostructure allowing for their ability to focus light into small volumes and thus generate large enhancements in the amplitude of the local electromagnetic field [61]. This resonant interaction is also used to perform chemical reactions. Hot electrons, in the context of photocatalysis, can holistically be defined as electrons with energies >> *kT* above the Fermi level on optically excited plasmonic nanoparticle surfaces that are then transferred to a medium (be it a chemical adsorbate, a semiconductor, or even the surrounding environment) where they perform a particular function (in photocatalysis, a chemical reaction). In this manner, plasmonic photocatalysis allows for the manipulation of light with nanometer-scale precision, and for reaction control of hot carrier processes at sub-femtosecond timescales [46]. Nearly all semiconductor-based hot electron photocatalysts demonstrated until now consist of Schottky junctions of *n*-type semiconductors with plasmonic noble metals (Figure 2). Figure 2 indicates that on the metal side, a fraction of electrons with energies exceeding the Schottky barrier height are able to cross over into the semiconductor side of the junction. Figure 2 shows that photogenerated holes in the semiconductor drift towards the metal because of the built-in field associated with the Schottky junction. There is a negligibly small equilibrium concentration of holes on the semiconductor side of the junction due to which the hot electrons that do cross over from the metal would be expected to have long lifetimes in the semiconductor, due to the lack of recombination events. In lifetime semiconductors, such as Si, Ge, InP, etc., charge neutrality will be restored in a duration roughly comparable to the dielectric relaxation time (<100 ns) [62]. In relaxation semiconductors, such as TiO_2_, ZnO, GaN, SrTiO_3_, etc., the dielectric relaxation times are orders of magnitude larger than in lifetime semiconductors due to which injected hot electrons can have unusually long lifetimes of milliseconds to even hours, which allows abundant time for these electrons to reduce reactant species [42,62,63,64]. Au/*n*–TiO_2_ plasmonic noble metal nanostructure–semiconductor heterojunctions are particularly ubiquitous. There is emerging interest in using *p*-type TiO_2_/noble metal heterojunctions to achieve enhanced photocatalytic performance by enabling the fast injection (and subsequent utilization) of hot holes into TiO_2_ before appreciable thermalization. The basic motivation for the use of such heterojunctions lies in the asymmetric energy distribution of hot carrier pairs produced through Landau damping of the particle plasmon. In Au, the hot carriers produced consist of high energy holes and low energy electrons, i.e., the hot holes are much hotter than the hot electrons, making it more worthwhile to drive chemical reactions using hot holes [65,66]. Anodization and sol-gel synthetic strategies that combine a high density of Ti^3+^ defect states together with elevated temperature oxygen annealing to reduce oxygen vacancies are known to produce *p*-type TiO_2_ [67,68]. Jinhua Ye and colleagues constructed a Schottky junction consisting of *p*-type TiO_2_ decorated with Au NPs and observed a remarkable fivefold enhancement of the acetone evolution rate in the photocatalytic degradation of isopropanol. Likewise, Y. Zhang et al. [69] observed a significant enhancement in the photocatalytic degradation of tetrabromobisphenol A using Ag-loaded *p*-type TiO_2_.

**Figure 3:**
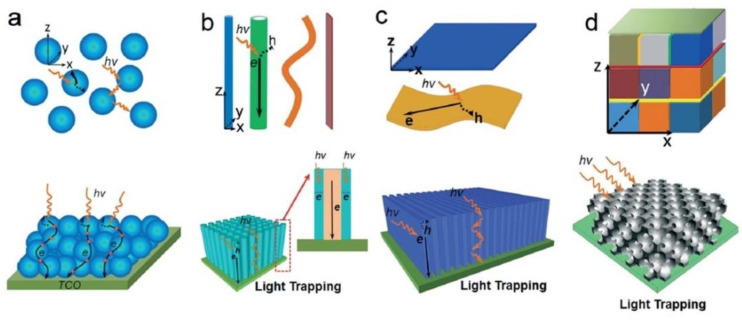
Various nanoscale architectures that can be used in photocatalytic applications from (**a**) 0D nanocrystals, (**b**) 1D nanostructures, (**c**) 2D nanosheets and films, and (**d**) 0D-1D-2D integrated 3D nanostructures. The figure also illustrates the light scattering, light trapping, and charge transport processes in the corresponding nanostructures. Reprinted with permission from Ref [9] Copyright 2015 Royal Society of Chemistry. Figure 3d originally adapted by Ref [9] from Ref [86] with permission from John Wiley, and reprinted here with permission from John Wiley. Copyright John Wiley and Sons (2013).

As mentioned earlier, when it comes to semiconductor photocatalysts, TiO_2_ remains the benchmark thanks to its relatively low cost, high availability, low toxicity, stability in both acidic and basic media, and resistance to photo-corrosion [29]. TiO_2_ has a wide band gap (3.2 eV for anatase and 3.0 eV for the rutile phase) and a relatively high absorption coefficient for ultraviolet photons [71]. TiO_2_ has a minority carrier (hole) diffusion length of 70 nm for anatase TiO_2_ and 10 nm for the rutile phase [72,73]. Due to its large bandgap, TiO_2_ primarily absorbs UV photons and taps into merely 5% of the solar energy that our planet receives. Although doping can extend the light absorption range of TiO_2_ from the UV to visible wavelengths, the absorption coefficient and the photocatalytic activity of TiO_2_ typically decrease [9,29,73,74]. To compensate for these limitations, various architectures of TiO_2_ photocatalysts ranging from powders in aqueous solutions [74], nanoparticles (0D), nanorods and nanotubes (1D), nanosheets and films (2D), and 0D-1D-2D integrated nanostructures (3D) (Figure 3) have been investigated [9]. 0D structures have the highest surface area per unit mass, a beneficial feature for catalysis, but have the disadvantages of not being able to sustain an internal electric field and confining both electrons and holes in a small volume of space until charge separation occurs. 1D structures combine a high surface area and the possibility of intra-nanowire charge separation due to a built-in field with the orthogonalization of light absorption/charge generation and charge separation processes [75,76]. Other efforts have focused on varying crystalline phase systems (rutile, anatase, and brookite) [77,78,79], doped heterojunctions, and mesoporous supports [80,81] with the focus being to optimize integral nanoscale properties, such as the optical path length, carrier mobility [82,83], charge carrier kinetics [84], light absorption [85], band bending, etc. Despite all of this, definitive success in sensitizing the photocatalytic activity of TiO_2_ to visible wavelengths is yet to be achieved.

This is where plasmonic photocatalytic systems incorporating the use of TiO_2_–plasmonic noble metal heterojunction nanostructures enter as a viable and realistic solution for extending the photoresponse of TiO_2_; hot electrons are at the core of this development. The family of plasmonic noble metals is small with gold (Au) and silver (Ag) being the two most recognized elements. Using the knowledge that Au and Ag nanostructures both have low loss surface plasmon resonances excited by visible and near-infrared photons, one can promote and mediate the charge transfer of hot electrons to the neighboring TiO_2_ semiconductor which can be utilized as a secondary surface or port for photocatalytic reactions in addition to photocatalytic reactions occurring on the surface of the noble metal. In a standard system, when placed in intimate contact, Au and *n*-type TiO_2_ form a Schottky junction. Noble metals have a high work function, and in the case of Au, its Fermi level is located below that of *n*-type TiO_2_. Upon contact, the Fermi levels equilibrate resulting in the bending of the conduction band of TiO_2_ and the formation of a Schottky barrier (Figure 2). Thus, a depletion region is formed, where an internal electric field is maintained and directed from TiO_2_ to Au. Upon excitation by incident visible light, it is this electric field that drives the motion of photogenerated electrons in the depletion region to move to TiO_2_ and holes to Au, thus preventing recombination. These electrons and holes are the ones to participate in photocatalytic reactions [30].

Plasmon-mediated electron transfer involving the injection of hot electrons across a Schottky barrier from Au NPs into the conduction band of TiO_2_ is known to occur at timescales of 250 fs or shorter [87,88], and the transferred electrons have been observed to exhibit unusually long lifetimes (>10^3^ s) in rutile TiO_2_, larger by two orders of magnitude than the lifetimes of photoexcited carriers generated directly in TiO_2_ [64]. The importance of nanostructured Au NP–TiO_2_ heterojunctions lies in the fact that hot electrons formed in Au NPs by decay of plasmons stimulated by visible photons with energies well below the bandgap of TiO_2_, have been demonstrated to drive chemical reactions subsequent to injection across the Schottky barrier into TiO_2_. Thus, heterojunctions of plasmonic nanoparticles with TiO_2_ enable visible light sensitization. The sensitization effect can be maximized in geometries where the plasmon-mediated local electromagnetic field enhancement at the metal–semiconductor interface is large, i.e., at hot spots [89,90].

A key question relates to the theoretical maximum power conversion efficiency (PCE) achievable in a plasmonic hot electron cell. White and Catchpole demonstrated that for a typical parabolic density of states (DOS) in the conduction band (CB) of the metal, the PCE was capped at 7.2%, rising however to 22.8% if the CB DOS could be engineered such that electrons close to the Fermi level (*E_F_*) were preferentially excited over lower energy electrons during the non-radiative decay of the particle plasmon [91]. This calculation assumed the sequential mechanism of plasmon decay and did not take into account more direct hot carrier formation and separation mechanisms, such as chemical interface damping. It is important to note that there are no lab-scale or commercially deployed photocatalysts that can convert sunlight into chemical fuels over extended durations with PCEs of over 5%. Therefore, while the PCEs potentially achievable using plasmonic hot electron devices might seem unremarkable for photovoltaics where lab-scale PCEs of 20–25% are routinely obtained for single junction silicon, CdTe, and halide perovskite solar cells, the same PCEs if achieved in the context of photocatalysis, would constitute a dramatic enhancement over the state of the art.

Au and Ag remain the most popular plasmonic noble metals in use. Ag is an ideal material for plasmonics, due to its low optical loss in the visible and NIR spectral ranges [92]. Au performs equivalently well in the visible and NIR spectral ranges and is also chemically superior to Ag which oxidizes under ambient conditions. Various other plasmonic noble metals have been considered in the field including Cu, Al, Pt, Pd, etc. Surface plasmons form at visible and near-infrared wavelengths in the base metals Al and Cu. However, the much larger dielectric losses (due to both radiation- and interband damping in Al [93] and interband damping in Cu [94]) result in broad, low quality factor resonances with a weak local field enhancement and insufficient production of usable hot carriers. Furthermore, Cu and Al are chemically unstable under atmospheric conditions. These reasons limit the use of Cu and Al to niche applications that exploit the LSPR resonances of Cu and Al in the IR and UV spectral ranges. Pd and Pt exhibit very strong interband damping [93], and have attracted attention in plasmonic catalysis, largely due to their catalytic abilities, and are often incorporated in bimetallic plasmon systems, due to their weak absorption at visible wavelengths. Beyond this, various studies have been conducted over the years to extend and diversify the library of plasmonic materials that could be utilized for plasmonic applications [95]. The alloying of different noble metals has been an alternative to tune the LSPR wavelength [96,97]. Similar approaches have also been considered in the fabrication of bimetallic and trimetallic systems where plasmonic noble metals are fabricated in conjunction with a catalytic metal with the former serving as a nanoantenna and the other as a catalytic medium [60]. Efforts have also been made to modulate the LSPR behavior of noble metals via exotic morphologies [98]. Atomistic and continuum calculations have provided deeper understanding of the plasmonic responses of these noble metals, and recent efforts have also focused on the use of phase and compositional changes to help evoke plasmon responses in lower cost, non-plasmonic noble metals, transition metal oxides and nitrides, and chalcogenide compounds [21,99,100,101,102,103].

The extraction of hot electrons from plasmonic nanoparticles would be useful in a variety of applications, including cancer tissue targeting [104,105,106], lasing [107,108], imaging [109,110], molecular characterization [51,111,112], and solar energy conversion (solar cells and photovoltaics) [113,114,115,116]. Some of these applications focus on the design of plasmonic nanostructures that optimize the confinement, bending, and propagation of light while minimizing internal losses, while others focus on charge carrier formation and transfer processes [117]. Plasmonic photocatalysis applications fall in the latter category. In fact, Au nanoparticles coupled to TiO_2_ for water splitting constitute the earliest examples of plasmon-enhanced photocatalytic and energy conversion systems. It is critical to understand the inherent nature and origin of these microscopic high energy charge carriers for their efficient implementation in photocatalytic systems. Gaps in knowledge and understanding persist within the scientific community regarding the origin of hot electrons in optically excited plasmonic nanoparticles, and the subsequent charge transfer dynamics that take place within said systems to promote photocatalytic activity.

Our objective is to shed light on this issue by providing a comprehensive review of the knowledge we have thus far gleaned on hot electrons. These discussions are supplemented by an extensive outlook on how to characterize the dynamics of these transient and highly energetic charge carriers through exotic spectroscopic techniques, and their direct use for experimental progress in the field of plasmonic photocatalysis for various applications. A perspective on future work, theoretical and experimental alike, that may further assist in elucidating the true nature of hot electrons and plasmonic phenomena is also provided in the closing sections.

## 2. Digging Deeper into Hot Electrons

Early seminal research on semiconductor devices, and the physical modeling of extended metal surfaces set the foundation for our current understanding of hot electron phenomena. The term “hot electrons” not only describes the individual electrons themselves, but also describes the Fermi–Dirac distribution of electrons in a solid albeit with an elevated effective temperature—the effective temperatures involved when considering the carrier kinetic energies and carrier densities in the solid, and not that of the solid itself- as opposed to thermal equilibrium [118].

### 2.1. Surface Plasmons

**Figure 4:**
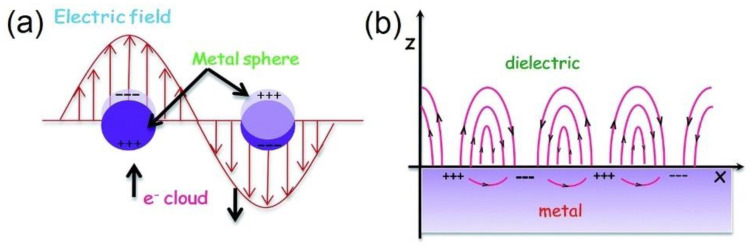
Schematic illustrations of (**a**) Localized Surface Plasmon Resonances and (**b**) Surface Plasmon Polaritons. Note the differences in morphologies of the structures involved. LSPRs are excited on metal nanostructures smaller than the electron mean free path within the material as well as smaller than the wavelength of incident light, such as the nanospheres in (**a**) where free electrons are displaced from the positive ions, driven by the propagating electric field component of the incident light, and oscillate collectively in resonance. In (**b**) the metal surface’s characteristic dimension is larger than the wavelength of incident light resulting in the excitation of a propagating surface plasmon polariton that travels along the surface with evanescent waves that diminish perpendicular to the surface. Reprinted with permission from Ref [120] Copyright Royal Society of Chemistry (2016).

Hot electrons are now a major focus in the field of plasmonics, which is the study of the interaction of light with free electrons in a metal. Hot electrons are generated via surface plasmon (or plasmon) excitation. Surface plasmons are the quantized collective and coherent oscillations of free electrons in a metal in response to excitation by incident photons at a metal-dielectric interface [119]. The electric field of the incident light guides the collective oscillations of these free electrons resulting in two characteristic modes: Surface Plasmon Polaritons (SPPs) and Localized Surface Plasmon Resonances (LSPRs). These resultant modes are largely determined by the morphology of the metallic structures that enable them (Figure 4) [30].

The excitation of SPPs occurs predominantly in continuous metal structures with characteristic dimensions larger than the incident wavelength of light. The corresponding plasmon oscillations propagate primarily along the metal surface for distances of tens to hundreds of micrometers, while declining as evanescent waves perpendicular and away from the metal surface [121]. In the opposite extreme, for metal nanostructures that are smaller than the electron mean free path within the material and the incident wavelength of light, LSPRs are generated [30]. Here, the collective oscillation of the free electrons at the metal-dielectric interface is driven by the electric field of the incident light with a resonance being achieved when the frequency of the incident radiation matches the oscillation frequency of the free electrons in the metal nanostructure. While SPPs are representative of propagating or traveling plasmons, LSPRs are characterized by non-propagating or standing wave plasmons confined strictly within the boundaries of the metal nanostructure. LSPRs can be excited on metal nanoparticles of various geometries including spheres, prisms, cubes, shells, etc., as well as around nanoholes or nanorods or nanoscale corrugations in thin metal films [122].

The resonant interaction observed in LSPRs is the main factor towards the confinement of photonic energy to the surface of the nanostructure for a duration that exceeds the time-scales photons would spend in the same volume traveling at the speed of light [123]. Consequently, there is an amplification of the local electric field of the incident light as well as the formation of a high concentration of energetic electrons at the surface of the nanostructure. In conclusion, plasmons can be understood as charge density oscillations (non-propagating LSPRs or propagating SPPs) that are a result of dipole and higher order multipole formation in the metal structures described above, due to incident electromagnetic wave excitation.

### 2.2. Sequential Mechanism of Hot Electron Relaxation

Hot electrons proliferate various critical technologies that take advantage of the LSPR mechanism. In the context of plasmonic photocatalysis, the focus is largely on LSPRs in plasmonic nanoparticles rather than SPPs, unless mentioned otherwise. LSPR excitation can be used to drive remote and direct photochemistry; photonic energy can either be transferred to nearby semiconductors, metals, and molecular photocatalysts or facilitate chemical transformations that occur directly on the surface of the plasmonic nanostructures [123]. All these processes are characterized exclusively by electron or hole transfer from excited metal nanoparticles to acceptor states in semiconductors or molecules. In explaining the charge transfer dynamics involved, the scientific community has, for a large part, been guided by the well-established work on the physical modeling of extended metal surfaces.

**Figure 5:**
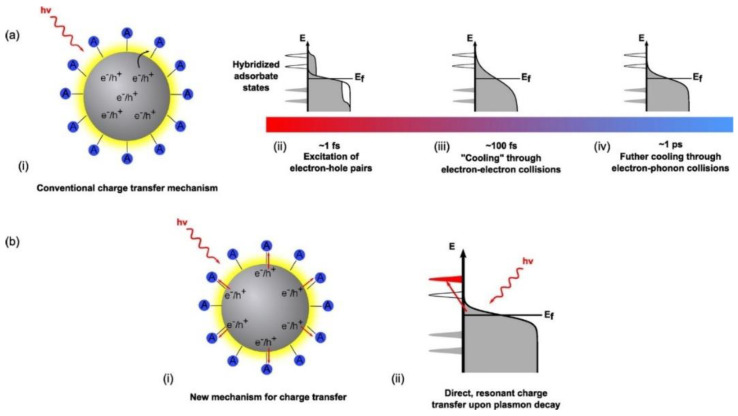
Modes of charge transfer and relaxation mechanisms in metal nanostructures. Ref [117] In the conventional charge transfer mechanism (**a**), resonant photon absorption creates hot electron-hole pairs within the metal nanostructure. What begins as an equilibrium thermal distribution of charge carriers in the metal nanostructure rapidly changes (i) to a nonequilibrium athermal hot electron distribution (~1 fs) (that cannot be described by Fermi–Dirac statistics) (ii) Hot electrons are now continuously transferred to the conduction band of the semiconductor from the tail portion of the electron distribution of the noble metal (iii) This athermal distribution rapidly dephases or cools through electron–electron collisions taking place on the order of ~100 fs. (iv) Further cooling through electron phonon collisions occurs on the order of ~1 ps resulting in the thermalization of the initial athermal distribution and a subsequent relaxation towards equilibrium. Alternatively, in (**b**) there is the Dissociation Induced Electron Transfer (DIET) mechanism, where electrons generated under excitation are directly injected into the conduction band of the semiconductor without and before any further interactions with other electrons. (ii) It is a direct, resonant transfer of charge carriers that circumvents the thermalization and relaxation mechanisms of hot electrons depicted in the sequential mechanism in (**a**). Reprinted with permission from Ref [117] Copyright American Chemical Society (2016).

Conventional theory suggests a sequential charge excitation/transfer process (Figure 5a) to explain charge transfer dynamics when LSPR excitation occurs on a plasmonic metal nanostructure. Here, a photon excites the LSPR of a nanoparticle to form plasmons, which dephase nearly instantaneously to yield excited electron-hole pairs. The non-radiative decay of plasmons into electron-hole pairs can involve either intraband transitions or interband transitions [124]. A competing mechanism is the radiative decay of particle plasmons. Dephasing refers to the reduction in the amplitude of coherent motion of electrons, which in turn depends on the strength of the coupling of the plasma oscillation to the electron-hole continuum [125]. Dephasing that involves the loss of energy from the collective oscillation of free electrons to the excitation of individual electron-hole pairs is also known as Landau damping [126]. In ~20 nm sized nanoparticles formed by electron beam lithography, single particle near-field scanning optical microscopy (NSOM), and second harmonic generation (SHG) spectroscopy have been used to measure dephasing times of 4–8 fs in Au NPs and 7–10 fs in Ag NPs [125,127]. The electron-hole pairs produced in a few femtoseconds, due to dephasing, are distributed over a range of energies allowing higher energy charge carriers to occupy acceptor states in nearby semiconductors or molecules. The subsequent cooling of the energetic electron-hole pairs to yield a Fermi–Dirac distribution of electrons at an elevated temperature occurs over tens to hundreds of femtoseconds with contributions from electron–electron scattering in the bulk, radiative damping, and electron-surface collision damping (Figure 5a) [61]. Further relaxation of the carrier distribution occurs through collisions with phonons over a few picoseconds, as shown in Figure 5a, while the excited phonons equilibrate over hundreds of picoseconds. A kinetic representation of these damping processes is used in time-resolved studies involving femtosecond pulse experiments where electrons are observed to be excited via low-energy and high-energy transitions. The distribution of electrons during the femtosecond-pulse would include a large population of low-excitation energy electrons and some high-excitation energy electrons. Considering the relaxation time scales involved in the excitation of the LSPR, where electron–electron scattering occurs in ~100 fs [128,129] followed by electron–phonon coupling at the picosecond timescale, the conventional description of LSPR excitation on a plasmonic nanoparticle says that there are significantly few highly excited electrons in the final thermal electron distribution compared to the initial excited distribution.

As a consequence of LSPR excitation, two types of charge carriers can be distinguished: (i) low-excitation energy charge carriers (also identified as Drude electrons and holes) near the Fermi level, and responsible for plasmon oscillations, and (ii) high-excitation energy charge carriers (the “hot electrons”) with energies >> *kT* above the Fermi level. The prerequisite for plasmonic photocatalysis is the efficient extraction of these hot electrons to help support or drive photocatalytic reactions but, according to the conventional theory stated above, the expected yield of hot electrons is low. This is because a large fraction of the formed energetic charge carriers lacks sufficient energy to support photocatalytic activity or energy transfer reactions, and most of the charge carrier energy is immediately lost upon LSPR dephasing through interactions with other electrons and phonons within the nanoparticle.

### 2.3. Alternative Mechanisms of Hot Electron Relaxation

In the sequential excitation and relaxation picture, the key to efficient plasmonic photocatalysis lies in extracting hot carriers before they fully equilibrate. However, there have been multiple experimental observations reporting fundamental deviations from the conventional description of the charge excitation/transfer mechanism, most notably concerning semiconductor-to-adsorbate charge transfer reactions. The extraction process involves tunneling through or thermionic emission of the hot carriers over the Schottky barrier into the semiconductor, as shown in Figure 2. Considering the number of hot carriers with sufficient energy and momentum to cross the barrier, the nature of the carrier distribution, the probability that hot carriers will reach the semiconductor-noble metal interface and the transmission probability across the interface, the sequential mechanism dictates that injection efficiencies of ~1% are expected [130]. However, hot electron injection efficiencies of 20–50% for Au NP−TiO_2_ NP heterojunctions have been observed by multiple research groups using femtosecond transient absorption spectroscopy [130,131]. The sequential excitation-relaxation/transfer picture also requires hot carriers to be extracted from the metal by the semiconductor at timescales shorter than a few hundred femtoseconds. Such ultrafast charge transfer at timescales of 50–240 fs following excitation using 550 nm photons (close to LSPR of gold spheres) has indeed been observed in Au NP−TiO_2_ NP heterojunctions [132,133].

**Figure 6:**
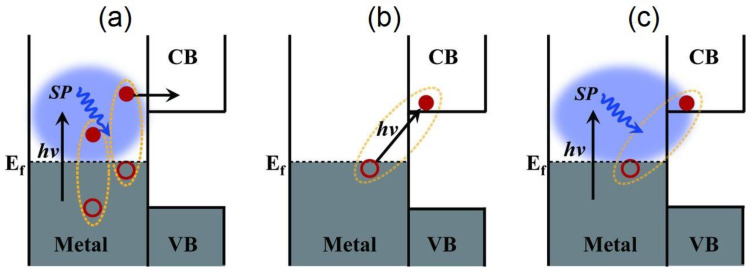
A perspective on the various charge-separation pathways in noble metal–semiconductor systems. (**a**) The conventional plasmonic hot electron transfer (PHET) mechanism where a plasmon (blue ellipsoidal cloud) in the noble metal dephases into a hot electron-hole pair via Landau damping, following which, the hot electron is injected into the conduction band (CB) of the semiconductor. The electron-hole pairs generated in such a manner display a broad distribution of energies. (**b**) The IFCT mechanism where an electron in the noble metal is directly excited into the CB of the semiconductor, and its plasmonic counterpart in (**c**) PICTT where the plasmon dephases with the direct creation of an electron in the CB of the semiconductor and a hole in the metal. VB indicates the semiconductor valence band, while *hν* is the energy of the incident photon. Reprinted with permission from Ref [134] Copyright The American Association for the Advancement of Science (2015).

A completely different mechanism for hot electron harvesting has been suggested involving the direct excitation of interfacial charge transfer (IFCT) states (Figure 6b). The IFCT mechanism refers to plasmon induced metal-to-semiconductor interfacial transitions (PICTT) [134] where it is postulated that the noble metal plasmon as well as the strong coupling and mixing of metal and semiconductor levels allow for the direct generation of an electron in the semiconductor and a hole in the noble metal (Figure 6c). While plasmon-induced IFCT was demonstrated in Au NP−CdSe heterojunctions [134] and has long been implicated in the anomalously high visible response observed in nanostructured Cu–TiO_2_ heterojunction photocatalysts [135,136,137,138,139,140,141], direct excitation of charge transfer states is more commonly found in experiments with the addition of adsorbates to the surfaces of plasmonic nanostructures. It is well known that molecule-to-semiconductor electron-transfer reactions can occur at sub-picosecond timescales [142]. This is primarily due to the high density of vacant acceptor states in the semiconductor and invocation of Fermi’s golden rule. Contrastingly, the reverse charge-transfer reaction from semiconductor-to-molecule has been shown to be quite slow, due to the much lower density of states in the molecules [143]. Therefore, the possibility for a significant amount of hot electron transfer from the noble metal to vacant molecular states, according to the conventional theory described above, would be highly unlikely.

Surprisingly, multiple experimental observations have reported that the presence of a chemical adsorbate on a plasmonic nanoparticle can indeed lead to even faster relaxation of the LSPR over timescales of ~5 fs [123]. This relaxation of the LSPR induced by the chemical adsorbate is generally referred to as chemical interface damping (CID). CID describes how the addition of adsorbates, absent in the IFCT mechanism, to the surfaces of plasmonic nanostructures induces a broadening of the plasmon band, while providing another direct and additional pathway for the dephasing of the plasmon. Irrespective of the conflicting timescales for electron transfer from metal nanoparticles to a chemical adsorbate and the opposite case alike, excitation of an interfacial charge-transfer transition has still been observed. The difficulties in reconciling this new ultrafast relaxation time with the indirect sequential mechanism described in the conventional theory has led to the development of a contending explanation where molecule plasmon-enhanced photocatalysis reactions are suggested to proceed through a more direct mechanism or a “dissociation induced by electronic transitions” (DIET) process (Figure 5b) [61,117,123,144]. In this direct excitation mechanism, a charge-transfer transition is directly excited such that the prior processes of internal relaxation of electrons and damping of the LSPR within the metal nanoparticle are seemingly irrelevant. Furthermore, the DIET process has been used to explain the high quantum yields for hot electron charge transfer observed in plasmon-induced oxidation reactions involving resonant photo-induced electron transfer from Au and Ag nanoparticles to strongly bound molecules or semiconductor quantum dots [61,117,144]. DIET is a subset of CID describing small molecule plasmon-enhanced interfacial charge transfer processes where the excitation of the charge transfer transition transiently occupies a surface bound anionic state of the adsorbed molecule. Conventional theory describes this state to be vibrationally excited, and to relax rapidly through vibrational cooling followed by electron transfer back to the metal, but when the excitation rate overcomes the relaxation rate, dissociation can be activated resulting in reactive species that promote photocatalytic reactions [61]. The distinction between CID and electron transfer following plasmon decay remains an unsolved mystery. It is also unclear if CID and electron-surface scattering are distinct phenomena [145]. In the context of the present review, DIET is highly relevant to those cases where an organic molecule is adsorbed on the noble metal–TiO_2_ heterojunction such as: the oxidative degradation of organic compounds using TiO_2_–Au photocatalysts [70], the vapor phase reduction of CO_2_ over TiO_2_–Au NP photocatalysts [146], the photocatalytic oxidation of CO over Au NP–TiO_2_ [147], and the use of reduced graphene oxide (rGO) bridges to shuttle hot electrons more effectively between Ag/Au NPs and TiO_2_ [148,149].

A fundamental difference between the direct and sequential mechanisms revolves around the question of when plasmon-dephasing exactly occurs. The sequential mechanism describes plasmon dephasing to occur before electron-transfer reactions, whereas in the direct transfer mechanism, it is the charge-transfer reaction itself that leads to plasmon dephasing. The importance of this distinction between the two processes has signified the necessity for further research on CID processes to examine how molecules interact with electrons in metal nanoparticles as well as the need to identify the source and exact microscopic origin of the plasmon-induced hot electrons. Lee et al. [150] have explored possibilities towards controlling, tuning, and optimizing the contributions provided by CID as a channel for plasmonic hot-electron energy transfer. Using scanning electron microscopy-correlated dark field scattering, Lee et al. [150] studied the electronic nature of CID behavior for systems of benzene adsorbates on gold bipyramids with similar aspect ratios to those of gold nanorods. The bipyramidal morphology of the gold nanostructures alongside the electronic effects of the adsorbate molecules were observed to generate increased interfacial contact between the plasmonic noble metal and molecular adsorbate systems. As such, electron withdrawing groups on the adsorbates were found to induce larger homogeneous and high quality LSPR line widths as opposed to those of electron donating groups that experienced a weakened LSPR response due to back transfer of electrons. Lee et al. [150] demonstrated that CID in the LSPR can thus be tuned by controlling the electron withdrawing and electron donating features of the molecular adsorbates deposited on the surface of a plasmonic noble metal. Using a complementary approach, Foerster et al. [145] have demonstrated that CID scales inversely with the effective path length of electrons, in other words, the average distance of electrons to reach the surface, in the plasmonic noble metal. As such, they pose a resultant study demonstrating that by modifying the characteristic dimensions of the noble metal, i.e., gold nanorods of different sizes (14 × 41 nm, 18 × 55 nm, 22 × 66 nm, 27 × 78 nm) but similar aspect ratios, one can moderate the contribution of CID in comparison to other competing plasmon decay channels with CID becoming the dominating plasmon energy decay mechanism, in their work, via the observation of increased plasmon line width broadening for very small gold nanorods.

In the context of plasmonic photocatalysis, the sequential mechanism and the IFCT mechanism are used to describe and postulate the charge transfer mechanisms involved in plasmonic noble metal–semiconductor heterojunctions. Au and Ag are the preferred candidates as plasmonic noble metals, while TiO_2_ serves as the electron-accepting *n*-type semiconductor [151]. As shown in Figure 2, visible light absorption by the Au/Ag nanoparticle results in collective oscillations of *sp* band electrons, and the creation of hot electrons in the *sp* conduction band. These hot electrons dephase rapidly resulting in poor hot electron injection efficiencies. In order for successful hot electron injection to occur, the hot electrons as well as the resultant hot holes must satisfy a number of conditions: the charges must be able to reach the surface of the plasmonic nanoparticle, and from there have enough energy (above the metal’s Fermi level) to overcome the Schottky barrier, while the residual hot holes must be extracted to maintain charge neutrality within the plasmonic noble metal [152]. The charge separation process must conserve both the energy and momentum of carriers. The hot holes and electrons formed by this charge separation at the metal–semiconductor interface help initiate oxidation and reduction reactions, thus promoting plasmonic photocatalysis. Moving forward, it is necessary to combine the knowledge aggregated from the postulated theories as well as existing experiments to further probe and improve our understanding of hot electron phenomena so that we may harness the advantages they provide for plasmonic photocatalysis applications.

## 3. Probing Hot Electrons

A variety of spectroscopic methods have been utilized to probe hot electron phenomena. These methods span select ranges of the electromagnetic spectrum and are distinct from one another in the differing modes of characterization they offer. This section will provide an overview of hot electron characterization methods along with brief summaries of their applicability.

### 3.1. Photoemission Spectroscopy

**Figure 7:**
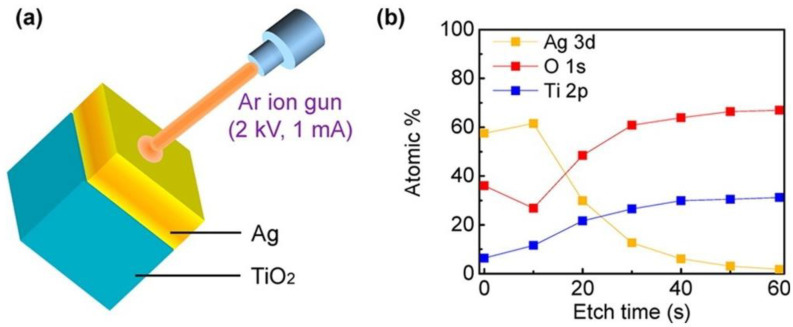
Schematic of XPS depth profile characterization. XPS and UPS are both based on the photoelectric effect, where an incident X-ray or UV photon of energy *hν* is absorbed by an atom resulting in the emission of a photoelectron. This photoelectron is of binding energy *E_b_* and is ejected with a kinetic energy *E_k_,* such that *E_k_ = hν − E_b_ − φ*, where *h* is Planck’s constant and *φ* is the work function. In (**a**) the spectra of a 10 nm thin layer of Ag on a TiO_2_ film is measured during etching using an Ar ion gun (at 2 kV and 1 mA) 6 times at intervals of 10 s, the result being a map of the depth profile (**b**) of the Ag film on the TiO_2_ layer where the atomic compositions are displayed in percentile measures along with their energy level occupancies [154]. Reprinted with permission from Ref [154] Copyright American Chemical Society (2014).

Photoemission spectroscopy is based on the photoelectric effect where a sample exposed to incident light of sufficient energy higher than the Coulombic binding energies of electrons to the nucleus emits electrons. The ejected flux and energy of the emitted photoelectrons depend respectively on the intensity and wavelength of the incident light. X-ray Photoelectron Spectroscopy (XPS) and Ultraviolet Photoelectron Spectroscopy (UPS) are common photoemission spectroscopy methods utilized in probing hot electron phenomena. Both methods assist in studying the kinetic energy distribution of the emitted photoelectrons and help provide information on the elemental composition and electronic state of a given surface. XPS has been used prominently in catalysis to provide information on the elemental composition (Figure 7), and the oxidation state of elements on the sample surface. This is accomplished by exciting the sample using soft x-rays to examine the core energy levels of the sample. Meanwhile, UPS utilizes UV radiation to examine valence energy levels, and is most suitable to probe bonding in metals and adsorbed species, while also providing a measure of the macroscopic work function of the given surface analyzed [153].

**Figure 8:**
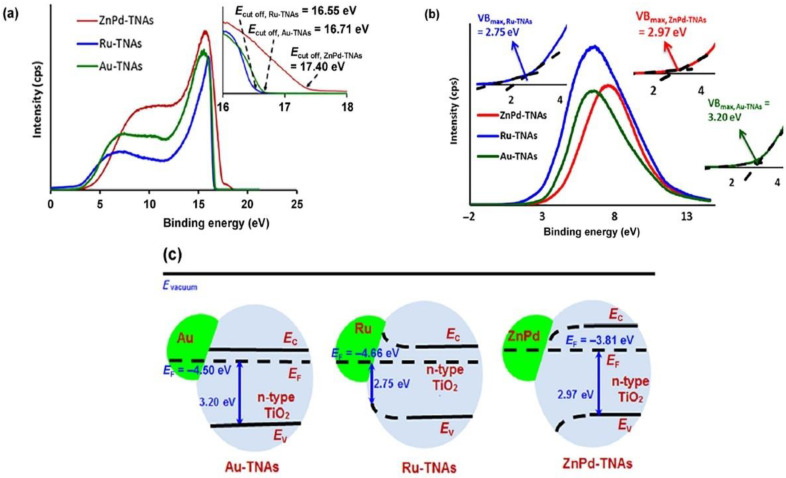
Kar et al. [155] utilized UPS to characterize band energetics in their work on enhanced CH_4_ yield via photocatalytic CO_2_ reduction using TiO_2_ nanotube arrays (TNAs) grafted with Au, Ru, and ZnPd nanoparticles (NP). In (**a**) the work functions of Au-TNA, Ru-TNA, and ZnPd-TNA are extracted to equal 4.50, 4.66, and 3.81 eV, respectively. In order to determine the positions of the valence band maxima for each structure, (**b**) UPS high binding energy cut-off spectra are utilized with cut-off energies at 3.20, 2.75, and 2.97 eV in Au-TNA, Ru-TNA, and ZnPd-TNA, respectively. The importance of these measurements is illustrated in (**c**), where the band structures of the noble metal–semiconductor composites are elucidated. Since a He laser of incident energy 21.21 eV was utilized, the work function can be calculated from the expression 21.21—E_cut-off_, where E_cut-off_ is the cut-off energy. Given the earlier values found in (**a**,**b**), the band-bending at the NP-TNA interfaces is measured. Thus, the UPS spectra assist in the significant observation of the differing band bending dynamics that occur in TNAs in contact with Ru NPs (upward bending) and TNAs in contact with ZnPd NPs (downward bending). This is particularly helpful in facilitating hypotheses and discussions on the charge transfer dynamics that may occur in such composite systems involving metal NP co-catalysts on metal oxide semiconductor supports, and their subsequent use as potential photocatalysts for a variety of chemical reactions. Reprinted with permission from Ref [155] Copyright Springer Nature (2016).

In considering hot-electron phenomena, UPS and XPS measurements provide for intricate knowledge of the energy structure of the given surface including measures of the metal’s work function, built-in fields, the kinetic or bulk energy distribution of ejected electrons (which translates to providing a distinction between low-energy Drude electrons and high-energy hot electrons following decay of the LSPR where electron-hole pairs are distributed over a range of energies in the metal’s electronic bands), and the density of states. UPS is also important when it comes to the study of adsorbates as UPS spectra of adsorbed species help reveal the binding energies of electrons in the orbitals of the adsorbate [153]. This is extremely significant when addressing the concept of DIET where hot electrons are transferred directly to a chemical adsorbate on the surface of the metal nanoparticle and promote subsequent catalytic reactions; in said scenarios, UPS can help image the hybridized metal-adsorbate states (bands) that are formed and analyze interfacial charge transitions (Figure 8).

**Figure 9:**
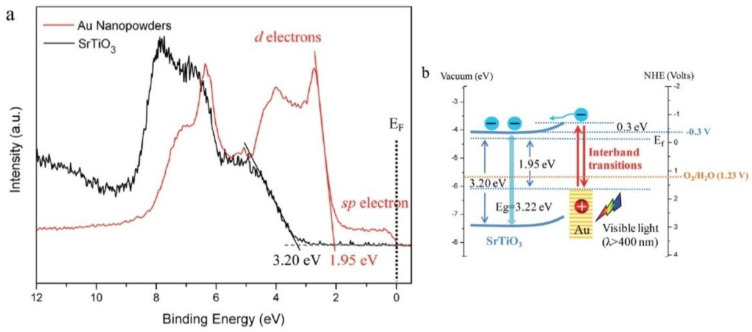
Applying XPS to a gold photosensitized SrTiO_3_ system used for visible-light water oxidation via Au interband transitions [158]. (**a**) The use of XPS allows for the measurement of the oxidative potential of holes leaving the plasmonic metal (Au) during interband transitions, and provides a confirmation of the valence band maximum of SrTiO_3_ at 3.20 eV below the Fermi level. The band edge of the Au nanopowders can also be identified at 1.95 eV (5d- band edge) with the tail edge attributed to 6sp electrons. This helps in the construction of the band energy diagram of the plasmonic system in (**b**), where the CB minimum of SrTiO_3_ is around −0.3 V vs. NHE with the expected band bending after contact (0.3 eV); a basic illustration of the use of XPS methods to illuminate the energy structure of a given surface including measures of the energy distributions and potentials of the charge carriers involved. Reprinted with permission from Ref [158] Copyright Royal Society of Chemistry (2014).

Similarly, XPS has also been used to survey hot electron effects in wide plasmonic areas [156,157] while also providing insights into the evolution of the energy band structure of the Schottky junctions that are widely used in plasmonic photocatalysis (Figure 9).

**Figure 10:**
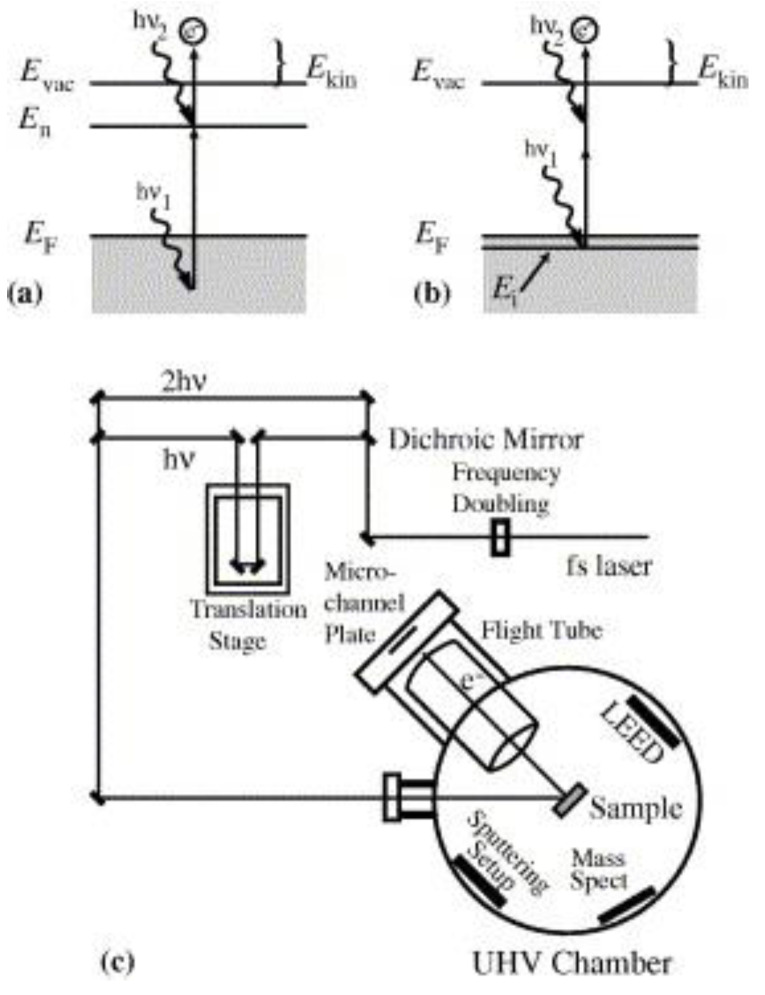
The Two-Photon Photoemission (2PPE) process. (**a**) Energy diagram for 2PPE of an unoccupied initial interfacial state. Absorption of photon 1 helps populate an excited intermediate state with a hot carrier and the absorption of photon 2 provides additional energy for the hot carrier to escape above the vacuum energy level. (**b**) The 2PPE process applied for an initially occupied interfacial state. (**c**) Schematic of the 2PPE experimental apparatus using a tunable femtosecond laser. Reprinted with permission from Ref [159] Copyright Elsevier (2005).

**Figure 11:**
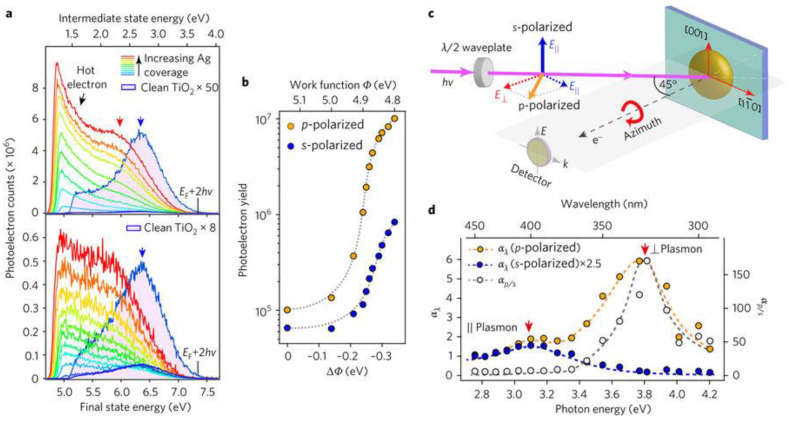
Characterization of Heterojunction Plasmons. (**a**) Deposition of Ag onto TiO_2_ is shown to enhance 2PP yields and consequently modifies the spectra of the *s-* and *p-*polarized excitations. The work function of the sample is noted to decrease around 0.35 eV, shifting the onset photoemission energy. (**b**) The 2PP yields are determined by integrating the photoelectrons counts with respect to the final photoelectron energy *E_f_* and are plotted as a function of the change in the work function, which is dependent on the depth of Ag coverage. (**c**) A schematic portraying the enhancements of the 2PP yield with respect to the incident laser wavelength polarization and crystal azimuth orientation. *p*-polarized light has both the parallel and perpendicular electric field components, while the *s*-polarized light consists only of the parallel component. (**d**) Wavelength dependence of the 2PP enhancement by the parallel and perpendicular plasmon modes at energies 3.1 and 3.8 eV. This is determined by taking the ratio of *p-* to *s-*polarization yields (right *y* axis) and Ag/TiO_2_ to Mo (a polycrystalline Molybdenum sample that assumes a flat spectral response) yields. Reprinted with permission from Ref [161] Copyright Springer Nature (2017).

Another suite of techniques that can directly provide information regarding the inelastic decay times of plasmonic hot electrons consists of time-resolved and spectroscopic two photon photoemission (TR-2PPE and 2PPE). In noble metals and noble metals containing heterojunctions, the sequential absorption of two photons (pump and probe respectively), each with energy below the work-function of the metal, is used to generate photoemission in 2PPE. Absorption of the first photon populates an excited intermediate state with a hot carrier while absorption of the second photon provides additional energy to the hot carrier over and above the vacuum level, thus liberating it as an emitted photoelectron whose energy is measured Figure 10. By changing the time delay between the pump and the probe pulses (TR-2PPE), energies of the emitted photoelectrons correspondingly change and the dynamics of hot carriers in the excited state can be observed [159]. It is critical in TR-2PPE experiments to create excited state populations of less than 10^−3^ carriers/unit cell in order to eliminate contributions toward inelastic decay from hot carrier–hot carrier scattering so that the time domain decays are characteristic of scattering of the hot carriers by cold electrons below the Fermi level [160]. This is achieved by limiting the intensities of the pump and probe pulses to the low µJ cm^−2^ range. Tang et al. [161] formed Ag nanoparticle islands on monocrystalline rutile TiO_2_ surfaces by electron beam evaporation and then used 2-PPE to probe hot electrons in the resulting heterojunctions. Tang et al. [161] discovered strong coupling between the 3.03 eV excitonic transition of TiO_2_ and the Mie-type surface plasmon in the Ag NPs, resulting in direct generation of hot electrons in TiO_2_ due to dephasing of the surface plasmon caused by chemical interface damping (Figure 11). The same study also found a rutile crystal plane dependence on the hot electron generation as well as an anisotropy (depending on optical polarization parallel or perpendicular to the substrate) in the hot electron distribution.

### 3.2. Auger and Electron Energy Loss Spectroscopy

**Figure 12:**
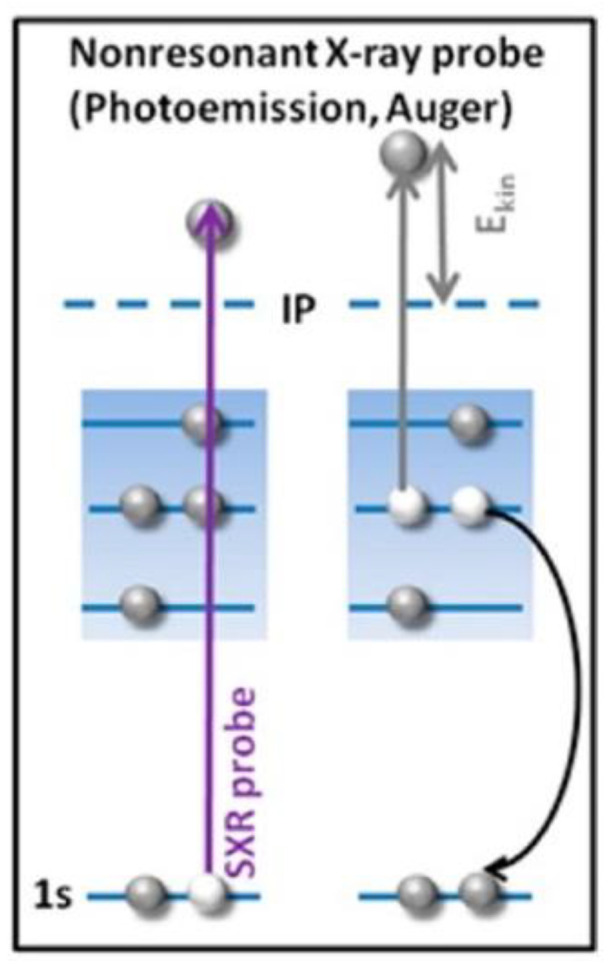
The Auger Effect, where we begin with a high-speed electron that knocks off an electron in the inner shell of an atom [157]. This leaves a vacant state (a 1s core hole), that is either filled by an upper electron that drops down to the inner shell, emitting a photon in the process (for heavy atoms, this energy is in the X-ray region, and thus results in X-ray fluorescence) or the excited ion relaxes by filling the core hole with an electron from a higher energy level, the resultant energy of this transition is taken up by an outer electron ejecting it from the atom, the Auger electron. The same is observed in the schematic where in non-resonant Auger spectroscopy, these vacancies are produced due to bombardment of a given sample with high energy electrons, in this case, a non-resonant X-ray pulse. Reprinted and adapted with permission from Ref [157] Copyright American Chemical Society (2016).

Auger Electron Spectroscopy (AES) is a heavily used spectroscopic method in the fields of materials and surfaces sciences [162,163]. While it is primarily applied in studies of film growth and surface elemental analysis, AES is also useful for understanding the electronic structure of atoms, and electron interaction and relaxation processes in solids or interfaces. The AES mechanism involves exciting a sample with a beam of primary electrons of energy between 1 and 10 keV [153]. AES is considered an electron-core level spectroscopy, and thus observes the relaxation of an excited atom via the transition of an electron from a higher shell to a core hole generated via the primary electron excitation. Energy released in this manner results either in x-ray fluorescence or the emission of a second electron, also called the Auger electron (Figure 12). AES is used correspondingly with Electron Energy Loss Spectroscopy (EELS). EELS can be considered as a subset of spectroscopic methods that deal with inelastic electron scattering and is often categorized as a vibrational spectroscopic method. In EELS, a beam of monochromatic, low-energy electrons is incident on a surface, resulting in excitations of lattice vibrations of the substrate, molecular vibrations of adsorbed species, and electronic transitions [162,163].

The energy spectrum of electrons resulting from a surface that is irradiated with a beam of primary electrons can provide for fundamental information on the surface’s electronic structure and the interactions that take place particularly with regards to the differing energy loss processes that the incident electrons can experience. These may include vibrations and electronic transitions, plasmon losses involving the excitations of the sea of electrons, and other inelastic processes. While AES provides for high spatial resolution and chemical sensitivity, several limitations are encountered when evaluating solid specimens. The most common of these limitations are charging effects in non-conducting samples [164,165]. This is when the number of secondary electrons leaving the sample is not equal to those incident, resulting in a net positive or negative charge on the surface. These surface charges can alter the electron yield emitted from the sample and distort the observed Auger peaks. The characteristic energy losses evidenced by electrons incident on a sample can also cause an obscuration of AES data. These losses appear mainly in the form of multiple scattering events, and collective electron density oscillations, i.e., plasmons [165,166]. This is identified by the fact that plasmon peaks, representing plasmon losses, can easily dwarf Auger peaks. Since Auger spectra are generally weak, and spread over a broad range of energy values, this makes it difficult to extract the spectra in the presence of plasmon losses. Thus, it is very common to have supplementary analyses through techniques like XPS to help in correctly identifying the Auger peaks [165]. Auger spectra can also help identify cascade ionization events, identified as “satellite” peaks or plasmon gains, hinting at chemical bonding that may be occurring at the relevant surface. The presence of said satellites can also be another source of distortion of the Auger peak.

Despite these weaknesses, AES is still a widely used surface analysis technique in chemical and nanostructure characterization. In fact, the very nature of these losses can be used as a measure of identifying plasmonic gains and losses in media. AES and EELS can serve complementary purposes in their utilization for probing plasmon relaxation or losses and plasmon gains or excitation processes that can occur in plasmonic metal-nanoparticle Schottky junctions [157]. Berenyi et al. [167] present work along similar lines where by combining results from EELS spectra and that of AES/XPS, they are able to provide for the distinction of two excitation mechanisms, “intrinsic” and “extrinsic” in Ge Auger spectra. The intrinsic process is referred to the creation of a core vacancy in the solid, while the extrinsic excitation is identified as the outgoing Auger electron that modifies the equilibrium potential in the solid. Both these processes contribute to the energy loss in the structure, thus distorting the resultant Auger spectra, effects that can be alleviated and distinguished using the complementary EELS spectra, which only provide for extrinsic plasmon peaks.

**Figure 13:**
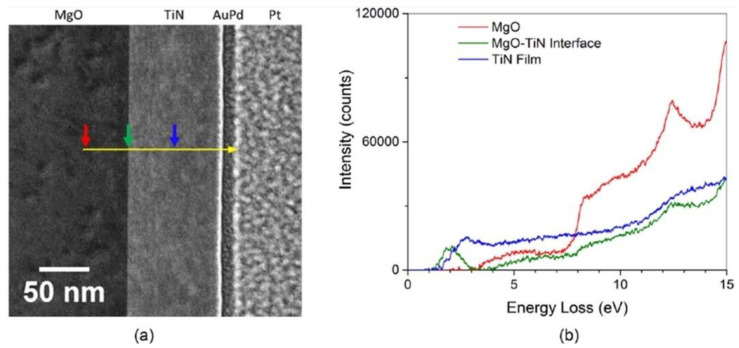
Using a combination of EELS with Scanning Tunneling Electron Microscopy (STEM) high-angle annular dark field (HAADF) imaging, Herzing et al. [168] determined the plasmon resonance characteristics of refractory TiN thin films. The spectra were collected by traversing (**a**) the yellow line from the MgO substrate through the TiN thin film, and to the opposite protective Pt layer. The spectra (**b**) are integrated over ten pixels at the locations of each colored arrow and indicate the local inelastic scattering distribution at said locations. From this, the spectral features typical of the MgO substrate are noted with an increase in inelastic scattering at 7.5 eV. At the interface of the MgO, and TiN film, a sharp peak due to surface-plasmon scattering is observed. A bulk plasmon resonance is identified at 2.81 eV and a weaker surface plasmon resonance peak was detected at 2.05 eV. The results are further supplemented by comparisons to finite difference time-domain simulations based on the measured optical data, which provide bulk and surface plasmon resonances with reasonable agreement at 2.74 eV and 2.15 eV, respectively. Reprinted with permission from Ref [168] Copyright Elsevier (2016).

EELS is an alternative method that can be used for the direct characterization of the plasmonic response of a material [168]. Generally, this can be carried out using optical techniques but diffraction in far-field methods and tip-dimensions in near-field techniques limit the spatial resolutions that can be achieved. This becomes a significant problem in detailed mappings of plasmon modes or measuring local variations within a material due to changes in structure or chemistry [169]. Herzing et al. [168] present EELS as the solution to tackling said problems while obtaining the plasmon resonance characteristics of refractory TiN thin films (Figure 13).

Similar work by other groups, including Colliex et al. [170] and Forcherio et al. [171], have proven the relevance of AES and EELS methods in analyzing and characterizing plasmonic nanostructures. Quantitative analyses involving spectra of individual metals and the observation of core hole–conduction electron coupling and plasmon creation via electron scattering have also been achieved [172,173]. With hot electrons, AES and EELS can provide information on chemical elements on the surface of a solid, the relevant binding energies, the density of states of the valence band, etc. [174] Furthermore, energy loss spectra can provide information on the discrete excitation-relaxation processes that occur upon LSPR dephasing, concerning nanoscale systems as opposed to established work on extended metal surfaces, further shedding light on hot electron mechanisms and the timescales involved [167].

### 3.3. Absorption and Photoluminescence Spectroscopies

**Figure 14:**
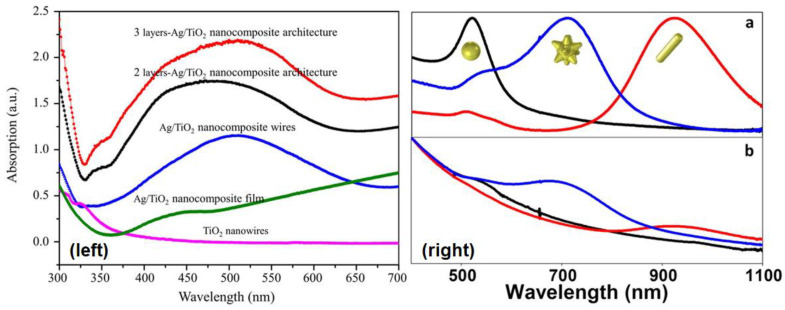
One application of plasmonic photocatalysis is to help extend the optical absorption capabilities of semiconductor photocatalysts, such as TiO_2_ (that largely absorb in the UV-Vis range) to visible photons. LSPR peaks in the visible spectral range for various cross-architectures of Ag/TiO_2_ plasmonic nanostructures (Left), as utilized in the work of Zhao et al. [176] Reprinted with permission from Ref [176] with attribution and adherence to Creative Commons Attribution License (CC BY) 4.0. Similarly, Castillo et al. [177] present (Right, (**a**)) the UV-vis-NIR spectra of free Au nanoparticles of varying structures from nanospheres (black), nanostars (blue), and nanorods (red), along with that of the UV-vis-NIR spectra of the same Au nanoparticles after they are adsorbed onto SiO_2_ beads following coating with TiO_2_ nanoparticles: SiO_2_@Au nanospheres@TiO_2_ (black), SiO_2_@Au nanostars@TiO_2_ (blue), and SiO_2_@Au nanorods@TiO_2_ (red). Thus, they are able to identify the unique absorption signatures of the three different morphologies along with a host of other properties including the locations of plasmon modes as evidenced by the peaks, and the fact that the UV-vis spectra of the composite structures (Right, (**b**)) display strong absorption bands at longer wavelengths. Reprinted with permission from Ref [177] Copyright American Chemical Society (2016).

**Figure 15:**
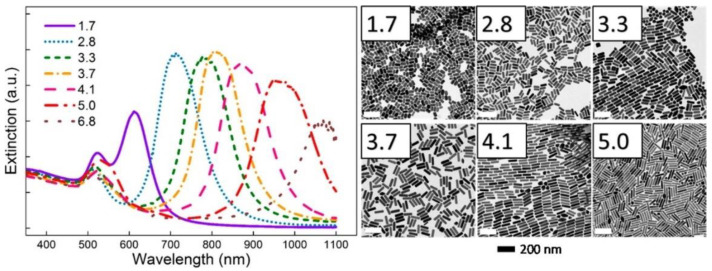
Another application of UV-Vis spectroscopy to characterize the dependence of plasmon resonance sensitivity on the geometry and morphology of the plasmonic system. UV-vis spectra of gold nanorods with aspect ratios varying between 1.7 to 6.8 along with the TEM images corresponding to each are shown. As is observed, the plasmon resonance of a gold nanorod can be tuned across the solar spectrum by controlling its nanogeometry. This has potential in the fabrication of composite, panchromatic plasmonic systems that ideally provide for broad and uniform absorption properties across the visible portion of the solar spectrum [178]. Reprinted with permission from Ref [178] Copyright American Chemical Society (2015).

Ultraviolet-Visible-Near Infrared (UV-Vis-NIR) spectroscopy is basically the study of electronic absorption. UV-Vis spectroscopy is widely used in analyzing hot electron phenomena as the resulting optical spectra detail the electronic structure, particularly the electronic excited states and their phonon replicas, in the given sample. UV-Vis spectroscopy is the simplest form of optical characterization of plasmonic substrates as it gives a direct measure of the extinction (absorption + scattering) behavior of the nanostructures involved as well as of the relevant wavelengths for plasmon resonance events (Figure 14 and Figure 15). These resonances are most often observed as peaks associated with the localized surface plasmon resonances of the sample at a resonance wavelength. The broadening of the LSPR peaks consists of contributions from homogeneous broadening due to the various processes producing efficient damping of the plasmon resonance as well as from inhomogeneous broadening that is due to local variations in the permittivity of the host matrix, non-uniformity in the nanoparticle size, and nanoparticle coupling effects [127]. The linewidths of the LSPR peaks of isolated plasmonic nanoparticles in UV-Vis spectra provide information regarding the timescales associated with damping processes, while the linewidths of ensembles of plasmonic nanoparticles also incorporate information regarding nanoparticle polydispersity and aggregation phenomena. Perner et al. [127] used Scanning Near-Field Optical Microscopy (SNOM) to measure the transmission spectra of single gold nanoparticles (mean diameter = 40 nm) embedded in a transparent TiO_2_ matrix with a dielectric constant of 2.2, and obtained the remarkable result that the peak wavelength for the near-field transmitted light intensity coincided with the peak wavelength of the far-field extinction spectrum of the ensemble of Au NPs embedded in the same dielectric matrix rather than with the absorption minimum of the Au NP ensemble. From this result, they were able to deduce that both the near-field transmission and the far-field extinction were plasmon mediated processes, except that the linewidth of the near-field transmission resonance of single Au NPs was purely due to homogeneous broadening, while the linewidth of the far-field resonance of the Au NP ensemble contained contributions from both homogeneous and inhomogeneous broadening mechanisms [127]. This insight enabled them to measure a linewidth of 0.17 eV for the homogeneous linewidth and ~0.17 eV for the purely inhomogeneous broadening. Klar et al. [175] used the homogeneous linewidth (Γ_hom_) of 0.18 eV obtained using SNOM studies of ~20 nm sized single Au NPs in a TiO_2_ matrix to estimate the surface plasmon dephasing time (given by *T*_2_ = 2ħ/Γ_hom_) to be 7 fs, and the local field enhancement (given by *f* = E_peak_/Γ_hom_) to be 10.8.

Electronic excitation of a conjugated molecule or semiconductor is followed by a relaxation process, termed photoluminescence, involving the spontaneous emission of a photon [179]. When photoluminescence occurs from singlet states over timescales of 0.01–100 ns, the emission is termed fluorescence, while phosphorescence involves light emission from triplet states, typically over timescales of 0.1–1000 μs. Both fluorescence and phosphorescence are strongly influenced by surface plasmons when present in close proximity to the emitter. On the one hand, the large free electron density in noble metal nanoparticles can quench the excited state and attenuate the emission of photons [180]. At the same time, the presence of an LSPR-amplified local electromagnetic field can induce a faster radiative decay of the excited state [180]. Surface plasmons have even been demonstrated to radiate the Franck–Condon structured emission of a molecular fluorophore, wherein the energy spacing between the vibronic energy levels remains similar to their separation in the molecule’s absorption spectrum [181]. Plasmon-to-exciton energy transfer mediated by Coulombic interactions (similar to Fӧrster-type resonance energy transfer in excitonic systems) can occur wherein the emission of the dye or semiconductor is enhanced (pumped) by the additional absorption associated with the plasmon resonance [182]. Yet another possibility is the hybridization of the plasmon and exciton to form a plexcitonic state, wherein splitting and shifting of the absorption and PL peaks are typically observed [183]. Which of these phenomena occur in a given semiconductor/noble metal NP system or dye/noble metal NP system, depends on the quality factor (*Q*) of the LSPR, the distance between the plasmonic nanoparticle(s) and the emitter(s), and the electronic coupling of the plasmon and exciton. If the plasmonic nanoparticle is in contact with the emitter, quenching of the excited state is expected to dominate. However, if the plasmon resonance has a high Q-factor and the distance between the plasmonic NP and the emitter is a few nm, an enhancement of the photoluminescence due to the local field enhancement can occur. In practice, the presence of a thin (1–3 nm) coating of an optoelectronically inert molecular monolayer or a dielectric spacer on the plasmonic NP can avoid the quenching of excited states while still permitting the interaction of the plasmon and the electronic excited state [184]. The presence of an unusually bright emission from the emitter at its normal emission wavelengths that is also accompanied by a much faster PL decay is a signature of plasmon-enhanced photoluminescence [185,186]. Thus, both steady-state and time-resolved PL spectroscopy are typically required to glean insights into the behavior of a plasmonic heterojunction. Apart from highlighting the inherent electronic and vibrational structure of a molecule, PL spectroscopy can be very effective in probing radiative and non-radiative decay channels and their corresponding lifetimes in LSPR relaxation processes [145,187,188].

**Figure 16:**
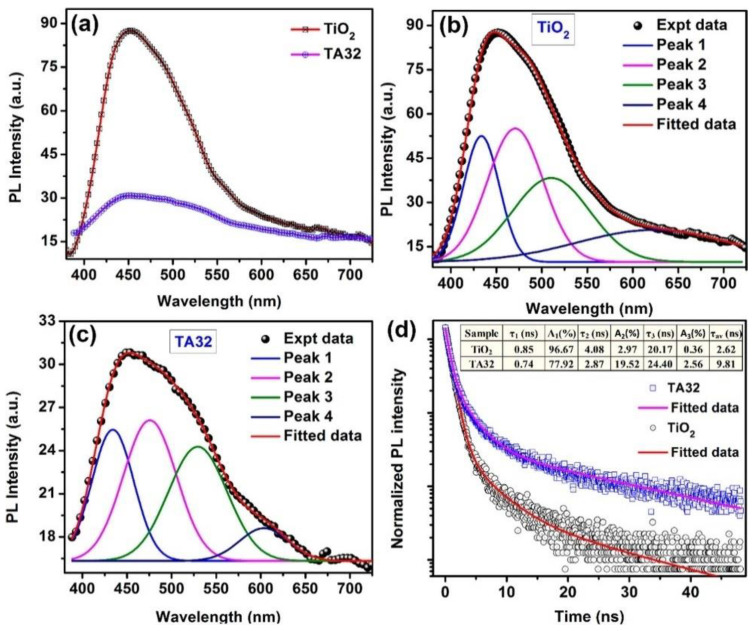
Photoluminescence is a useful technique to probe electronic interactions in plasmonic nanostructures, including the nature of defects, kinetics of charge recombination, and the migration of photogenerated charge carriers, as presented in the work of Paul et al. [194] (**a**) shows a comparison of PL spectra of pure TiO_2_ with that of the composite Ag–TiO_2_ excited using a 355 nm laser. It is noted that the PL intensity is highly reduced in the heterostructure, due to the introduction of Ag nanoparticles. Gaussian fitted PL spectra of TiO_2_ nanorods and the Ag–TiO_2_ heterostructure are respectively shown in (**b**,**c**). The PL intensity of the TiO_2_ nanorods is also seen to have decreased by ~3 times after decoration by Ag nanoparticles, while the PL spectra remain the same. By identifying the centers of the deconvoluted peaks in (**b**,**c**), Paul et al. are able to elicit the different characteristics of the given samples, such as self-trapped excitons at the TiO_2_ octahedra (Peak 1), shallow traps involving Ti^3+^ states below the conduction band (Peak 2), deep trap states associated with single electron trapped oxygen vacancies (Peak 3), and an intrinsic defect (Peak 4). Lastly, (**d**) provides a comparison of time-resolved photoluminescence spectra of pure TiO_2_ nanorods and the Ag–TiO_2_ heterostructure at 471 nm (emission) with 375 nm excitation. From this the lifetime of charge carriers in the different samples can be measured. Reprinted with permission from Ref [194] Copyright American Chemical Society (2017).

PL applied to observing hot electron phenomena would assist in probing the ultrafast relaxation processes of LSPR dephasing particularly with regards to interband and intraband scattering and transitions [60,189]. Note, knowledge of the type of transition, either interband or intraband, provides another means to distinguish between Drude and hot electron phenomena. PL also allows us to study the band structure of the relevant plasmonic structures considered, namely plasmonic metal–semiconductor Schottky junctions [189,190,191]. (Figure 16) The PL quantum yield of bulk metallic gold films under ultraviolet excitation is 10^−10^, which rises to 10^−6^ in spherical Au NPs, 10^−4^ in Au NRs and 4 × 10^−2^ in Au nanocubes [59]. The higher PL efficiency in plasmonic Au nanostructures is attributed to an accelerated radiative decay process due to the enhanced local field associated with the LSPR with sharp angled structures, such as nanoprisms, nanocubes, and nanostars, providing hot spots exhibiting the highest local field enhancements “lightning rod” effect. The emission mechanism in Au NPs is currently understood to involve an interband transition, namely the non-radiative recombination of *sp*-band electrons with *d*-band holes to emit particle plasmons that subsequently radiate [192,193].

**Figure 17:**
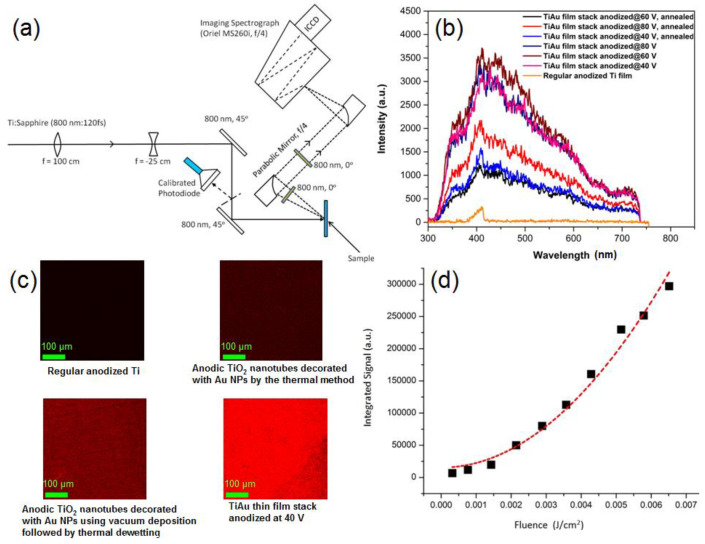
Two photon luminescence studies of Anodic Plasmonic Au–TiO_2_ Engineered Nanocomposites (A-PLATENs) [195]. The experimental setup (**a**) utilized by Farsinezhad et al. to obtain the (**b**) two-photon luminescence spectra from A-PLATENS. Two-photon luminescence images obtained (**c**) using a confocal microscope display the enhancement obtained with the novel A-PLATENs structures as compared to regular anodic titania nanotubes and anodic TiO_2_ nanotubes decorated with Au nanoparticles using conventional techniques, under identical excitation and imaging conditions. The two-photon luminescence intensity (**d**) as a function of fluence of an exciting laser for TiAu film stacks. Reprinted with permission from Ref [195] Copyright American Chemical Society (2017).

Two-photon luminescence (TPL) is a photoluminescence technique utilized to analyze the plasmonic response of noble metal nanoparticle systems [195,196,197] in which the noble metal nanoparticle is excited by the simultaneous absorption of two photons, usually in the infrared regime, with sufficiently intense laser illumination [198]. Single ultraviolet photon excited photoluminescence in plasmonic nanostructures exhibits a linear dependence of the emission intensity on the excitation power, and is understood to proceed through the recombination of *d*-band holes with *sp*-band electrons, as previously mentioned. Two-photon excited photoluminescence consists of both an up-converted broadband emission wherein near-infrared photoexcitation typically results in the emission of higher energy photons as well as a down-converted emission containing photons of higher wavelengths than the incident photon wavelength. The emission intensity in TPL is strongest near hotspots. The up-converted emission in TPL exhibits a near-quadratic dependence on the excitation power and has two sources—the first being interband recombination and the second being emission due to an intraband transition, namely the recombination of hot electrons within the conduction band. In some cases, the intraband recombination of hot electrons is the dominant TPL emission mechanism as evidenced by the similarity in the broadband emission of Au NP aggregates and Ag NP aggregates in spite of the marked different interband transition energies for Ag and Au [199]. In addition, the down-converted emission intensity has a linear dependence on excitation power. Furthermore, the PL lifetimes for one photon luminescence are of the order of tens of picoseconds and accompanied by blinking, while the TPL lifetimes are a few picseconds with no blinking present. In the case of a noble metal nanoparticle–semiconductor system, two photon luminescence studies act as a probe to analyze and image the plasmonic response of the system as well as explore the local dielectric properties of the media, and the resultant charge carrier dynamics. The majority of work in this area uses the two-photon luminescence response to measure the electromagnetic field-enhancement factor (EFE), image plasmon modes and spatially observe hot spots on the sample [200]. In principle, steady-state and time-resolved TPL could also be used to probe the dynamics of carriers created by interband transitions, specifically to understand whether photoexcited electrons in the several micrometer-thick noble metal–semiconductor nanocomposites are transferred to the semiconductor or not (i.e., by observing if some or all of the two-photon photoluminescence of the isolated noble metal nanoparticle is quenched in the presence of the semiconductor), and to measure the time-scales of competing processes, such as plasmon-mediated radiative recombination; however, very few reports have actively used TPL to examine phenomena specifically related to hot electrons. TPL was used by Farsinezhad et al. [195] in demonstrating the effectiveness of a novel plasmonic nanoparticle-embedded nanotube structure in generating a high local field enhancement that outperformed a plasmonic nanoparticle-decorated anodic nanotube structure. (Figure 17) Localized surface plasmon resonances, and surface plasmon modes are both influenced by the local dielectric properties, and fluctuations in the charge density of the given plasmonic material. These changes can often be observed via distinct changes in the photoluminescence characteristics of the materials, thus making two photon luminescence a practical technique to assess the plasmonic responses of said media [196,201]. Another variant of PL spectroscopy is Hot Photoluminescence (HPL) spectroscopy. HPL spectroscopy is a methodology that builds on the general idea of relaxation processes involving photoluminescence but focusing specifically on recombination processes of photo-created electrons with acceptor-bound holes [202].

### 3.4. Raman Spectroscopy

**Figure 18:**
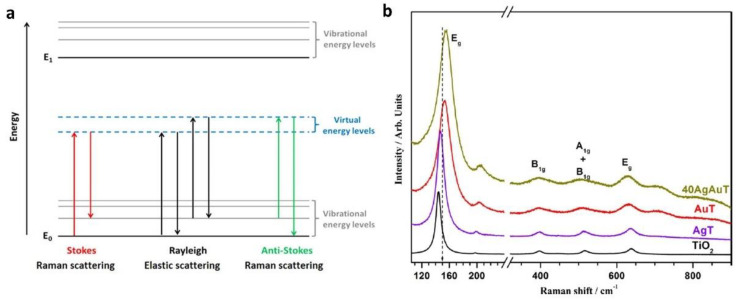
(**a**) Schematic of the Raman effect as utilized in Ember et al. [204] The shifting modes of inelastic scattering often evinced in Raman spectroscopy from the Stokes band (Left), where an inelastic vibration is excited to the anti-Stokes band (Right), where an already excited vibrational state is de-excited, the latter resulting in a higher energy photon being emitted as opposed to the former. Lastly, is the case of the Rayleigh band (Center) where the light scattered by the sample is done so without any loss of energy. Reprinted and adapted with permission and no modifications from Ref [204] with attribution and adherence to Creative Commons Attribution License (CC BY) 4.0. Copyright Nature Publishing Group (2017). (**b**) Raman spectroscopy as a characterization method. Raman studies of TiO_2_, Ag–TiO_2_, Au–TiO_2_, and Ag on Au–TiO_2_ composites. As discussed in Patra et al. [205], active Raman modes at particular wavelengths allow for the confirmation of the characteristic features of anatase TiO_2_, as well as the confirmation of SERS resulting in frequency shifts between SERS and normal Raman spectra of molecules observed in the peak shifts and broadening after deposition of gold on TiO_2_ for the differing composite systems. Reprinted with permission from Ref [205] Copyright John Wiley and Sons (2016).

Raman spectroscopy is a technique used to observe rotational, vibrational, and low-frequency modes in a system. Raman spectroscopy focuses on the inelastic scattering of monochromatic light incident on a sample, resulting in molecular vibrations, phonons, and other forms of excitation [203]. The molecular vibrations and systemic excitations because of Raman scattering can cause the energy of the incident photons on the sample to be shifted up or down. These shifting-modes of energy provide the relevant information regarding the electronic and vibrational structure of the system, and are categorized into three bands: Rayleigh, Stokes, and anti-Stokes bands (Figure 18). The Rayleigh band signifies elastic scattering (without loss of energy), while the Stokes and anti-Stokes bands relate to inelastic scattering where a vibration is excited with a down-shift in photon energy and scattering where a vibrational excited mode is de-excited with an up-shift in photon energy, respectively. The measurement of these signals arising from the Raman effect encompasses the technique that is Raman spectroscopy [153] (Figure 18).

When it comes to probing hot electrons, Surface Enhanced Raman Spectroscopy is a widely used technique. The SERS effect is fundamentally equivalent to the Raman effect except that it refers to the amplification of Raman signals from molecules adsorbed on metallic (typically noble metal) surfaces. The amplification of the signals in SERS is attributed to the electromagnetic interaction of light with noble metals resulting in amplification of the laser field through plasmon resonance excitations. The noble metals are in the form of metallic nanostructures ranging from colloids in solution to periodic elements on wafer substrates fabricated through nanolithography [119]. With SERS, the technique involves measuring the Raman signals of the adsorbed molecules which serve as the probes, which offers insight into the molecules’ electronic and vibrational structures [123] as well as help glean the interfacial nature of transitions occurring at the adsorbate-metal surface, which is essential in understanding hot electron processes in the context of plasmonic photocatalysis and DIET processes [206].

**Figure 19:**
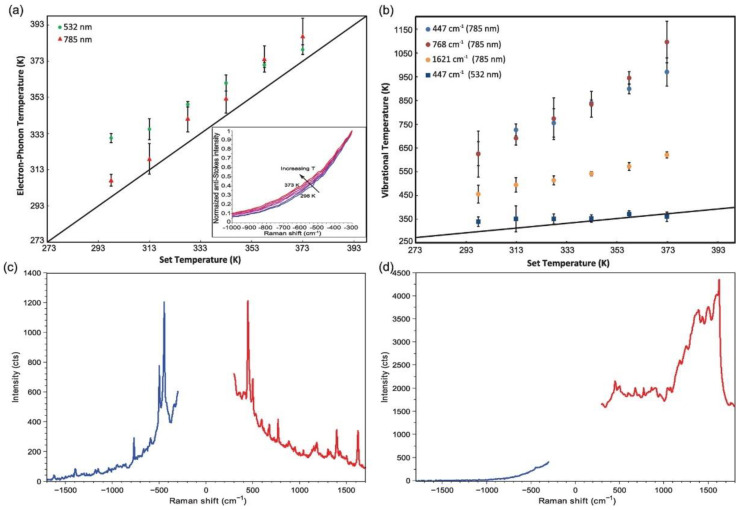
SERS as a probe to measure localized phonon temperatures of metal nanoparticles and vibrational adsorbate temperatures. Linic et al. [117] in (**a**) the temperature of the Ag nanoparticles under illumination by two laser sources (532 nm and 785 nm) are shown. Anti-Stokes spectra for the same is presented in the inset, along with temperatures of prominent vibrational modes of MB adsorbed on Ag (**b**), and the Stokes (red) and anti-Stokes (blue) spectra for the Ag–MB plasmonic system. There is a high anti-Stokes signal under 785 nm laser illumination (**c**) due to resonant charge transfer. The strong elevation in vibrational temperature of the adsorbed MB molecules under 785 nm laser illumination indicates resonant charge transfer from Ag to MB, while the similar temperatures of the Ag and the MB molecule under 532 nm laser illumination (**d**) indicates a lack of charge transfer, and a system in thermal equilibrium. Reprinted with permission from Ref [117] Copyright American Chemical Society (2016).

An example of the significance of SERS in analyzing hot electrons comes from the recent work accomplished by Linic et al. [117,144] In the vast literature on hot electron induced plasmonic photocatalysis, the focus has largely been on analyzing wavelength and intensity dependent Stokes SERS intensities of the adsorbed molecules on the plasmonic metal nanoparticles. Linic et al. were able to obtain unique insights by analyzing anti-Stokes intensities for a case study on methylene blue (MB) molecules adsorbed on Ag nanocubes. Using laser sources with photon wavelengths of 532 and 785 nm, Linic et al. used the anti-Stokes and Stokes SERS methods to simultaneously measure the vibrational temperature of the plasmonic nanoparticle and the attached adsorbate (Figure 19). This allowed them to track the flow of excited charge carriers in the system as the charge flow is directly related to the temperatures of the probe molecule and the plasmonic nanoparticles. Surprisingly, their experiments showed that charge transfer between the nanoparticle and the adsorbate took place under excitation by 785 nm photons as opposed to 532 nm photons, followed by selective heating of the molecule. This is a relevant result when one considers that the lowest energy of photons absorbed by free MB molecules is ~665 nm, providing further proof that the charge transfer is interfacial in nature (metal to molecule) as opposed to intraband transfers within the MB molecule. In an attempt to explain these charge transfer processes, Linic et al. were able to highlight the discrepancies in the conventional theory which would suggest higher rates of charge transfer for the 532 nm laser due to the higher energy photons involved as well as the higher extinction coefficient for the Ag SERS substrate at 532 nm compared to 785 nm. These results supplement other observations that have confirmed discrepancies in the application of the conventional theory of metallic surfaces applied to studies of LSPR phenomena [123,134]. Furthermore, the unique approach of using the anti-Stokes intensities to gauge these results is unprecedented, and only proves to signify the efficacy of SERS as an excellent technique to examine LSPR and hot electron transfer processes.

### 3.5. Kelvin Probe Force Microscopy

Hot electron injection plays a crucial role in photoelectric energy conversion and is a fundamental characteristic of plasmon-enhanced photocatalytic systems. Although studies involving photoluminescence quenching, and light-induced absorption spectroscopy have been used to study charge separation and transfer dynamics in plasmon-enhanced photocatalytic systems, these methods are limited by low resolution [207,208]. Kelvin probe force microscopy (KPFM), a noncontact variant of atomic force microscopy, allows for the mapping of surface potentials to study the structural and electronic properties of functional surfaces and interfaces at a nanometer-scale spatial resolution [209,210,211,212]. With KPFM, the work function of surfaces can be deduced at atomic or molecular scales. From the work function, various surface phenomena can be understood, including catalytic activity, doping, semiconductor band-bending, surface reconstruction, dielectric charge trapping and corrosion, etc. Recently, KPFM has been recognized as a versatile imaging technique that can be applied to plasmonic material/device investigations including perovskite solar cells, photocatalysts, and nanoscale biomaterials [213,214,215].

KPFM is a scanning probe method where the potential offset between a probe tip and a surface is measured. In KPFM mode, the tip is driven by the applied AC at the same frequency as the resonance frequency of the tip. While scanning the sample, the tip vibrates. The resulting vibration of the tip is detected using a setup that generally involves a diode laser and a detector. As the tip approaches the surface, a contact potential V_CPD_ is established and modulates tip vibration. The tip also has a direct voltage bias V_Bias_. These two values are used to determine the directive voltage:

ΔV_DC_ = V_Bias_ − V_CPD_

V_Bias_ is adjusted to be equal to V_CPD_ and achieve a V_DC_ = 0 in the KPFM feedback loop. In this manner, V_CPD_ becomes the difference between the surface potential of the sample and the potential of the tip,

V_CPD_ = V_Sample_ − V_Tip_

The relationship between V_CPD_ and the work functions of the sample and the tip can subsequently be written as [209,216],

V_CPD_ = (φ*_Tip_* − φ*_Sample_*)/*e*

According to this relationship, a smaller V_CPD_ corresponds to a higher sample work function.

**Figure 20:**
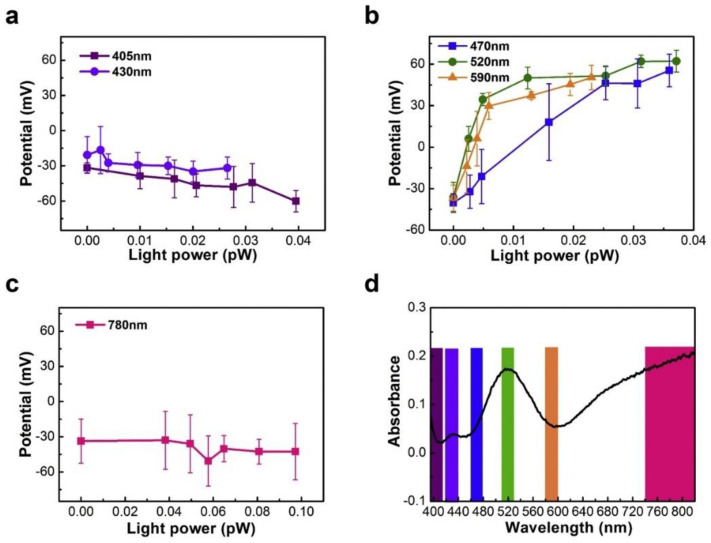
Using KPFM, Jian et al. [217] are able to study the modulation of the surface potential as a function of the light of power. (**a**–**c**) It is observed that with increasing light power toward the LSPR wavelength, more electrons are excited and flow to the Au nanoparticles resulting in a decrease in the surface potential of the Au nanoparticle. This switches upon the start of LSPR absorption resulting in a potential rise from −36 mV to 30 mV and gradual saturation at longer wavelengths. (**d**) Narrow band-pass filters of visible light for six central wavelengths: 405 nm, 430 nm, 470 nm, 520 nm, 590 nm, and 780 nm corresponding to characteristics of the absorption spectrum of Au nanoparticles (the dip, the low absorption peak, LSPR absorption, LSPR peak, end of LSPR absorption, and the long wavelength side). Reprinted with permission from Ref. [217] Copyright Elsevier (2019).

Jian et al. [217] demonstrate a working example of the KPFM for direct observation of hot electron injection at an Au–TiO_2_ interface. In their Au–TiO_2_ film, Au nanoparticles are sparsely distributed on the TiO_2_ film surface. Surface potential images of the Au–TiO_2_ sample under UV and visible irradiation is obtained using KPFM alongside AFM at the corresponding regions of interest. Through these simultaneous measurements, Jian et al. [217] are able to identify that the surface potentials of Au nanoparticles (alternating between dark and bright spots) are lower than that of TiO_2_ under UV irradiation, and higher under visible-light irradiation (Figure 20). The obtained surface potential differences indicate a steady-state concentration of hot electrons near the Au–TiO_2_ surface. This observation is consistent with the expected charge transfer mechanism in the Au–TiO_2_ system, which experiences a net generation, separation, and recombination of charge carriers. Upon visible light irradiation, hot electrons are generated in the Au nanoparticles due to the LSPR and migrate to the TiO_2_ film. This results in the formation of a weak current (I_SC_) in the Schottky barrier at the metal–semiconductor interface. As the hot electrons continue to migrate and accumulate, an electric field is formed that opposes the current of hot electron migration leading to a reverse current produced, due to the built-in electric field. As this reverse current gradually grows and becomes equal to the hot electron migration current, a potential difference is established across the interface. It is this potential difference that is studied using KPFM [217].

Among others, Jian et al. [217] also observe that the saturated surface potential follows the absorption spectrum of the Au nanoparticles. In this manner, KPFM is a versatile tool that can be used to observe charge transfer dynamics and phenomena at plasmonic interfaces. Lee et al. [218] demonstrate a similar run of experiments using the KPFM probe to directly observe plasmon-induced interfacial charge separation in metal–semiconductor hybrid nanostructures, where Au nanoparticles are attached to ZnO nanowires. They further extend this work to be successfully applied in characterizing plasmon–exciton or plexcitonic coupling between p-type poly(pyrrole) (PPy) nanowires and Ag nanoparticles. Apart from providing a means to observing the charge-transfer dynamics at plasmonic interfaces, Li et al. [219] have shown that KPFM can also be utilized to provide direct observations of the LSPR enhancements in plasmonic systems. Li et al. [219] use KPFM to observe the LSPR induced surface potential reduction in the vicinity of Ag nanoparticles on a GaN epilayer. Under UV irradiation, LSPR induced local field enhancements force photogenerated electrons to drift close to the Ag nanoparticles thus leading to a reduction of the surface potential around the nanoparticles. Similarly, KPFM mapping of the surface potential of Au nanoparticles on nonpolar ZnO and TiO_2_ nanotubes under UV irradiation have demonstrated greater understanding of the origins of the LSPR field enhancement and plasmonic interfacial charge dynamics [220,221]. These experiments support the growing support towards the use of KPFM for diverse experiments in understanding charge transfer dynamics, and LSPR field enhancement studies in plasmon enhanced photocatalytic and optoelectronic devices.

### 3.6. Other Prominent Methods

Beyond the spectroscopic methods discussed above, there remain many others that have been utilized in differing experiments on hot electron phenomena and plasmonic photocatalysis including: Infrared spectroscopy, Rayleigh light scattering [61,124,222,223,224], Ultrafast spectroscopies (Absorption, Photoelectronic, Transient Absorption) [225,226,227,228], angle resolved photoemission spectroscopy [229], time-domain terahertz spectroscopy [230,231,232], etc.

Analyses of hot electrons necessitate the integration of various spectroscopic techniques that are effective in extracting essential information on the dynamics of the ultrafast transient processes that accompany said phenomena. There are varying platforms and perspectives from which one could probe the microscopic origin of hot electrons. From time-resolved experiments that directly address the essential timescales involved in LSPRs (through Rayleigh Light Scattering, Transient Absorption spectroscopy, Terahertz spectroscopy) to experiments that focus on interfacial charge transfer dynamics (through Infrared and Two-Photon Photoelectron spectroscopy) [159,233,234], there are multiple angles from which the hot electron problem can be tackled. Having discussed the prominent spectroscopic methods utilized in examining this transient phenomenon, the following section will examine the construction and reported performances of realized hot electron photocatalytic systems.

## 4. Exploiting Hot Electrons

Numerous experiments have engaged the development of plasmon-enhanced photocatalytic systems that take advantage of high energy charge carriers or hot electrons [234,235,236]. A very typical hot electron harvesting system consists of a TiO_2_–noble metal nanoparticle heterojunction. Au and Ag are the most common plasmonic noble metals utilized in plasmonic photocatalysis applications, while TiO_2_ is a benchmark material for semiconductor-based photocatalysis [130,237,238,239]. In this section, we will briefly review hot electron phenomena in three categorical applications of photocatalytic technology: (i) photocatalytic degradation/aerobic oxidation of organic compounds, (ii) photocatalytic CO_2_ reduction and H_2_ generation, and (iii) photoelectrochemical water-splitting.

### 4.1. Photocatalytic Degradation/Aerobic Oxidation of Organic Compounds

**Figure 21:**
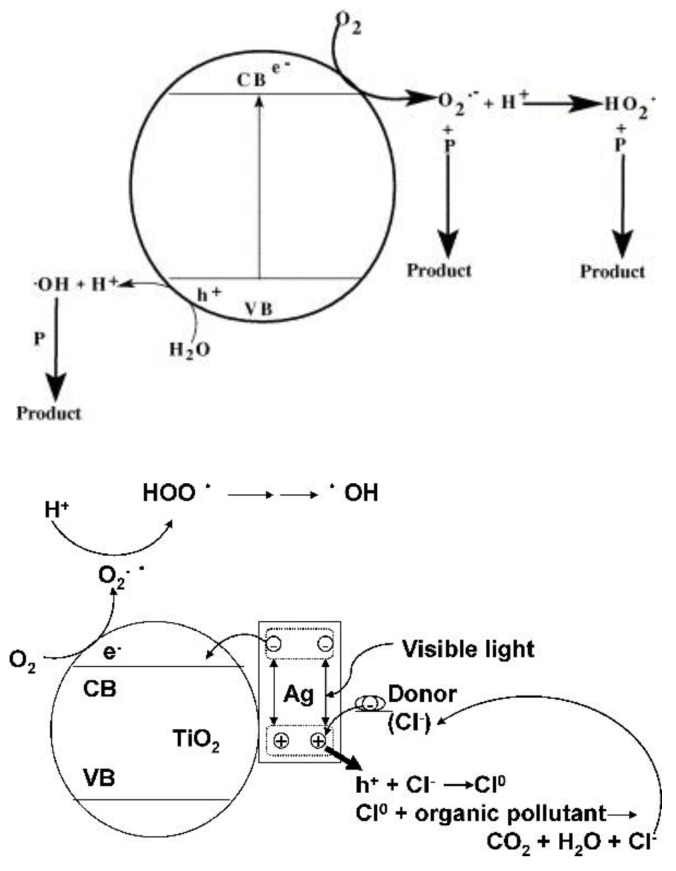
Photocatalytic degradation of organic pollutants [240]. In this case (Left), a suitable semiconductor, such as TiO_2_ can be used, the schematic presenting the oxidation of possible organic pollutants. The excitation of electron hole pairs results in their migration to the surface of the semiconductor, where oxidation reduction reactions can take place. Oxygen molecules capture electrons from the conduction band forming oxide radicals (O_2_*^−^*), which subsequently reacts with protons forming a hydroperoxide radical in (HO_2_*^−^*). Together these radicals assist in the degradation of organic pollutants. A similar notion of degradation is also evidenced on the side of the valence band, where holes are extracted. Reprinted with permission from Ref [240] Copyright Elsevier (2005). (Right) Schematic diagram of charge separation in a visible light irradiated Ag/AgCl/TiO_2_ system. Reprinted with permission from Ref [242] Copyright American Chemical Society (2009).

The efforts for photocatalytic degradation of organic pollutants follow the need to eliminate environmental pollution, which poses the risk of causing adverse effects to human health and the environment itself. Harmful chemical substances and pollutants can be emitted from various sources and permeate the environment for a long duration. Natural biodegradation of said pollutants is painfully slow and conventional treatment has been mostly ineffective and not environmentally compatible [240]. TiO_2_, notwithstanding its environmental friendly nature, is also an inexpensive and easily available photocatalyst that has proven to be very effective in breaking down organic pollutants and even achieve complete mineralization [241]. Furthermore, semiconductor systems are generally inexpensive, non-toxic, with highly tunable properties that can be adapted for a given scenario. Therefore, semiconductor photocatalytic degradation of harmful organic pollutants to non-hazardous products has been highly appealing in comparison to conventional chemical oxidation methods (Figure 21).

Noble metal nanoparticles are excellent catalysts for a variety of routine industrial scale high temperature chemical reactions, such as steam reforming, hydrogenation of olefins, aromatic ring opening reactions, etc. The basis for catalytic action in these systems is typically the coupling of phonon modes in the metal to vibrational modes in the molecules adsorbed on the surface of the metal such that the excess reorganization energy required for the transformation of the reactant to product is minimal [243]. However, such phonon driven catalysis allows very little room for engineering specific reaction products [113]. On the other hand, in hot electron mediated plasmonic photocatalysis, optically generated excess carriers in noble metal nanostructures can be used to engineer specific chemical transformations through DIET (Section 2.2) based on the energy of incident photons, the type of noble metal used, and the geometry of the plasmonic architecture.

TiO_2_, with a bandgap of 3.2 eV, is a photocatalyst that is only active within the UV spectrum of light. While UV irradiated TiO_2_ photocatalyzed degradation of organics has been feasible, considerable efforts have been made to modify TiO_2_ semiconductor systems to harness and extend the optical response of TiO_2_ to the more abundant visible source of light energy from the sun. Such alternatives have involved the use of dye-sensitization [244,245], selective substitutional or interstitial doping [246,247,248], and the incorporation of plasmonic metals in said semiconductor systems [97,249,250]. One example of a plasmonic system is Ag-loaded N-doped TiO_2_ photocatalysts [251] where a dramatic enhancement of visible-light induced photocatalytic efficiency was reported for Ag/N–TiO_2_ in the degradation of methyl orange (MO) due to a synergistic effect thanks to the incorporation of Ag-loading and N-doping. Characterization of the Ag/N–TiO_2_ was achieved using XRD, XPS, and UV-Vis DRS, the latter indicating a clear red-shift in the optical response of TiO_2_ photocatalysts along with higher visible absorbance in N–TiO_2_. It was inferred through the comparative data between N–TiO_2_ and Ag/N–TiO_2_ that the silver loading also promoted visible light absorption. Photocatalytic degradation activities of MO dye solutions under visible light irradiation by the photocatalyst for blank, TiO_2_, N–TiO_2_, and Ag/N–TiO_2_ nanoparticles showed a marked difference from negligible degradation to 8% MO degradation in TiO_2_, 37% MO degradation in N–TiO_2_, and up to 54% MO degradation in Ag/N–TiO_2_. A 0.5 wt% Ag content was observed to be the optimum amount for highest efficiency of the MO photodegradation for the N–TiO_2_ nanocatalysts. Schmuki et al. [252] have also observed that the attachment of Ag and Au nanoparticles on self-organized TiO_2_ nanotubular structures can significantly enhance UV-light induced photocatalytic decomposition of an organic pollutant in Acid Orange 7. Their work also highlights the need for a hierarchical structure that serves as a scaffold for the plasmonic nanoparticles with photocatalytic decomposition being more significant when loaded on nanotubular structures as opposed to compact TiO_2_ surfaces [252].

**Figure 22:**
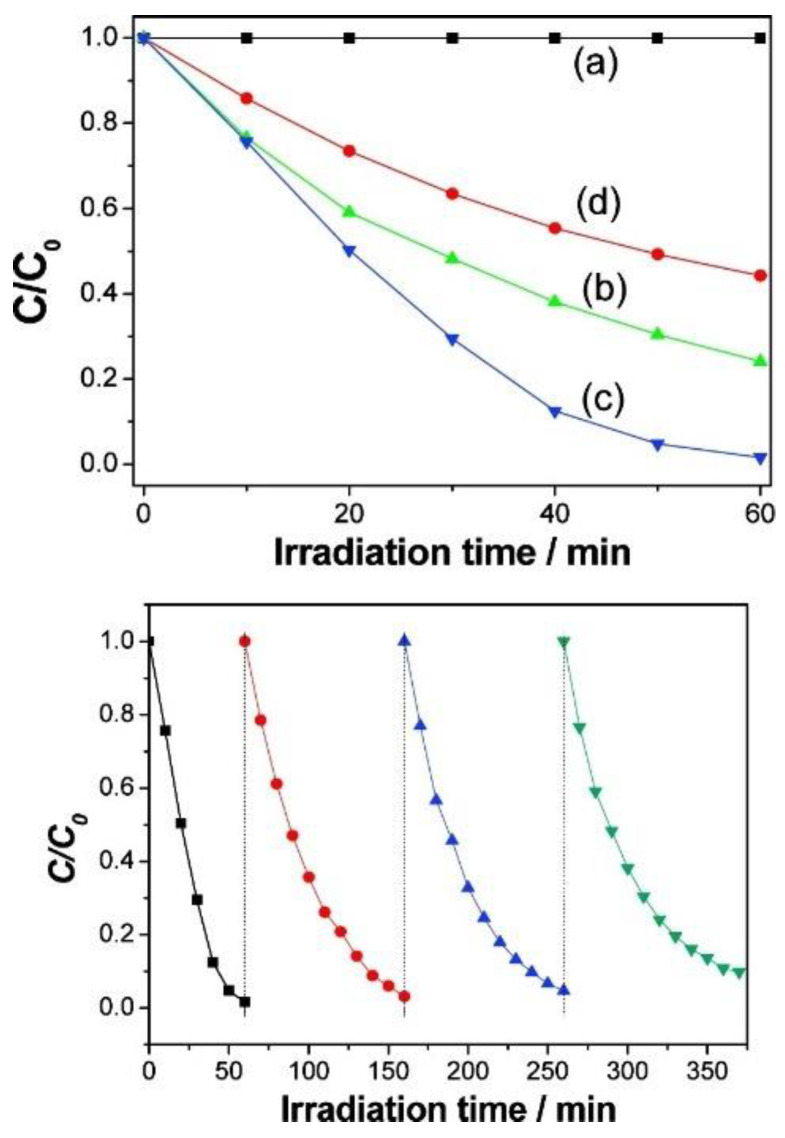
(Top) Comparison of photocatalytic activity and decomposition of Methyl Orange in Water of samples of (a) anatase TiO_2_ (b) amorphous Ag/AgCl/TiO_2_ (c) anatase Ag/AgCl/TiO_2_ and (d) anatase TiO_2−x_N_x_. (Bottom) Cyclic degradation curve for anatase Ag/AgCl/TiO_2_. Reprinted with permission from Ref [242] Copyright American Chemical Society (2009).

Similar results have also been observed in the application of Ag/AgCl/TiO_2_ nanotube arrays [242], the metal–semiconductor nanocomposite plasmonic photocatalyst exhibiting high visible-light photocatalytic activity for photocatalytic degradation of methyl orange in water (Figure 21). While comparing various compositions of TiO_2_ between amorphous and anatase phases under varying conditions of xenon illumination and thermal annealing, it was seen that the conglomerate of Ag/AgCl/TiO_2_ nanotubes expressed the greatest photodegradation activity and within a shorter period of time (60 min) as opposed to that achieved in the earlier work with 54% MO degradation in Ag/N–TiO_2_ in 120 min (Figure 22). The high visible-light photocatalytic activity and stability is attributed to the surface plasmon resonance absorption of silver nanoparticles under visible light irradiation, with charge separation occurring at the Ag NP–TiO_2_ hetero-interface where electrons transfer from plasmon-excited silver to TiO_2_ and from there to a donor in Cl to the silver nanoparticles.

In 2012, Tsukamoto et al. [253] formed plasmonic nanocomposites by decorating P25 TiO_2_ NPs (containing a mixture of anatase and rutile phases) with a layer of ~5 nm sized Au NPs. The formation process involved stirring a colloidal suspension of P25 NPs in an aqueous solution of chloroauric acid (HAuCl_4_) and subsequently calcining the colloids in air at elevated temperatures (typical calcination temperature: 673 K) [253]. The calcination process induced pyrolysis of HAuCl_4_ adsorbed on the surface of the TiO_2_ NPs to yield purple colored Au NP–TiO_2_ nano-heterojunction powders with broad LSPR peaks centered at ca. 550 nm. These Au NP-decorated P25 NPs were successfully used to aerobically oxidize a number of phenyl-substituted alcohols into their corresponding ketones with yields >80% under visible light irradiation containing photons of wavelengths >450 nm produced by a Xe lamp whose output was passed through a longpass optical filter [253]. For the oxidation of 1-phenylethanol into acetophenone, the Au NP-decorated P25 NPs produced four times as much acetophenone under visible light compared to the same reaction under the dark, and outperformed bare P25 TiO_2_ NPs by a factor of more than 7.5. The action spectrum for the aerobic oxidation of 1-phenylethanol closely followed the LSPR extinction profile and a maximum quantum yield of 8% at ~550 nm was reported. Interestingly, the Au NP-decorated P25 NPs performed significantly better than pure anatase NPs and pure rutile NPs decorated by Au NPs using the same process. Also interesting was the observation that 3.7–4.2 nm sized Au NPs formed by the HAuCl_4_ direct pyrolysis process dramatically outperformed 20–45 nm sized Au NPs formed using the photolysis of HAuCl_4_ (photodeposition) [253]. In 2014, wet impregnation of an aqueous solution of HAuCl_4_ into mesoporous anatase TiO_2_ with a pore size of ~11 nm followed by ultraviolet photolysis of the gold precursor was used by Bian et al. [254] in a highly cited paper to form nanoscopic TiO_2_–Au NP heterojunctions. Although the action spectra for photocatalysis obtained by Bian et al. closely resembled the LSPR profile seen in the optical spectra, very low maximum quantum yields were achieved, with an apparent quantum efficiency (AQE) of 0.05% for methylene blue degradation under illumination by 560 nm photons, and an AQE value of 0.11% for rhodamine B (RhB) degradation at the same wavelength [254]. Even lower quantum yields of ~0.0003% were obtained under visible light illumination for the photocatalytic generation of hydrogen from 50 vol% 2-propanol/water suspensions wherein the isopropanol served as a sacrificial hole scavenging agent [254]. By observing the electron accumulation-induced increase in the optical absorption of the TiO_2_–Au NP heterojunctions in the 600–1000 nm spectral range, Bian et al. clearly demonstrated that hot electrons in the Au NPs generated by the LSPR decay were injected into TiO_2_ [254]. Transient absorption spectroscopy revealed that the low photocatalytic quantum yields were due to fast (1–3000 ps) recombination of holes in Au NPs with trapped electrons in TiO_2_, even though the time scales involved in the hot electron injection process were comparable to or shorter than 80 fs. It must also be noted that the LSPR resonance observed by Bian et al. [254] was quite broad—implying a low Q-factor for the resonance, and the resulting weak local field amplification factor might have also contributed to the low quantum yields obtained. Hunsom et al. obtained a tripling in the rate of photoinduced glycerol oxidation by using Au NP-decorated TiO_2_ photocatalysts instead of bare TiO_2_ [255]. Further improvements in catalytic activity for glycerol photo-oxidation were achieved using bimetallic AuPd, AuPt, and AuBi NP/TiO_2_ heterojunctions with AuPd/TiO_2_ producing the highest performance which was attributed to optimal light harvesting and efficient charge separation [255].

There remains a continuing interest in the development and modification of TiO_2_–noble metal nanoparticle heterojunctions mainly due to their ideal application as photocatalytic systems in sensor, solar cell, photonic crystal, catalysis, separation technology, biomedical engineering, and nanotechnology [194,252,256,257,258]. As such, in recent years, differing structural schema of similar systems using plasmonic metals have been identified as viable for efficient photocatalytic degradation of organic pollutants [251,259,260,261].

### 4.2. Photocatalytic CO_2_ Reduction and H_2_ Generation

The development of sustainable, environment friendly, and renewable energy sources has been considered as a solution to the problem of managing the excessive amounts of CO_2_ in the atmosphere. These green strategies hold great economic significance [155], and assist in addressing the issues of carbon capture and our overwhelming use of fossil fuel energy. CO_2_ photoreduction is a green strategy that aims for the transformation of atmospheric CO_2_ and CO_2_ in exhaust emissions (e.g., flue gas from thermal power plants) into useful fuels and chemicals [6]. Conventional methods for CO_2_ photoreduction have ranged from biological (enzymes, algae, bacteria) chemical, electrochemical, and photochemical (visible light driven photocatalysis) systems. Unfortunately, several such systems suffer from a variety of shortcomings ranging from inability to control complex multielectron pathway chemical transformations, lack of mechanistic selectivity in reductive photocatalysis, low product yields, contamination, as well as lack of stability and reliability in design structure.

Here again, semiconducting nanomaterials have proven effective for the cause [16,262,263,264,265,266]. As before, many such systems incorporate the use of TiO_2_–noble metal nanostructure heterojunctions. Hou et al. formed 400 nm thick TiO_2_ films using a sol-gel process and then thermally evaporated gold on to the films to create discontinuous ~5 nm sized Au islands on the surface of TiO_2_ [146]. Annealing in air at 400 °C for four hours resulted in the formation of more spherical Au NPs, and the resulting Au NP–TiO_2_ heterojunctions exhibited a broad LSPR peak centered at ca. 565 nm. The plasmonic nanocomposites synthesized by Hou et al. [146] generated 22.4 µmol/m^2^-cat of methane from 51.6 mL of CO_2_-saturated water alone at a reaction temperature of 75 °C under 15 h illumination by the 350 mW output of a 532 nm green laser, a yield that was 24 times higher than that of bare TiO_2_ films under identical reaction conditions. The quantum efficiency for hot electron-driven plasmonic CO_2_ photoreduction was estimated to be 2.1 × 10^−5^% at 532 nm. The high selectivity for methane evolution under visible illumination was attributed to the band diagram of the Au–TiO_2_ photocatalyst vis-à-vis the reduction potentials of CO_2_/CH_4_, CO_2_/HCHO, and CO_2_/CH_3_OH. CO_2_/CH_3_OH is the only one of the aforementioned three reactions whose reduction potential the conduction band (CB) edge of TiO_2_ lies above of, thus restricting the activity of hot electrons injected from Au into the CB of TiO_2_, to initiating the methanation of TiO_2_ [146]. When 254 nm UV irradiation was used to illuminate Au NP–TiO_2_ heterojunctions or Au NPs on a non-semiconducting glass substrate while keeping all other conditions identical, the product mixture contained significant quantities of CH_4_, C_2_H_6_, HCHO, and CH_3_OH, indicating that the reactions occurred directly on the Au surface (whose Fermi level is lower than that of the TiO_2_ CB vs. the reduction potentials of CO_2_/CH_4_, CO_2_/HCHO, and CO_2_/CH_3_OH), stimulated by the interband transition in Au NPs [146].

Initially, efforts in this area focused on the use of systems incorporating only a single metal or a noble metal, but recently bimetallic nanoparticles containing at least one noble metal and loaded onto TiO_2_ have been found to be even more effective as photocatalysts. One such system is proposed by Kar et al. [155] in their use of TiO_2_ nanotube arrays grafted with Au, Ru, and ZnPd nanoparticles. The CH_4_ yields from CO_2_ photoreduction were 58.47, 26.37, and 26.83 µmol/gh for Au-, Ru-, and ZnPd TiO_2_ nanotube arrays, respectively. Kar et al. also demonstrated significant CO_2_ reduction activity for blue photons in the 420–480 nm spectral range. Jiao et al. formed hierarchical three dimensional ordered macroporous TiO_2_ (3DOM–TiO_2_) (macropore size: 200 nm) by sol-gel infiltration of the TiO_2_ precursor tetrabutyl titanate (Ti(OC_4_H_9_)_4_) into colloidal poly(methyl methacrylate) microsphere templates followed by subsequent hydrolysis to form inverse opals of titania [267]. This work provides yet another example of bimetallic nanoparticle decorated TiO_2_ surfaces for CO_2_ photoreduction since Jiao et al. decorated the surface of the titania inverse opals with 3–5 nm core-shell Au-Pd nanoparticles synthesized by the sodium borohydride reduction of HAuCl_4_ and PdCl_2_ in the presence of a poly(N-vinyl-2-pyrrolidone) (PVP) capping agent [267]. The Pd shell made the already broad LSPR peaks (λ_max_ = 550 nm) of the Au NPs even broader, so as to nearly obscure the LSPR profile, which is indicative of polydispersity in particle size and perhaps an additional damping mechanism for the Au particle plasmon involving carriers in Pd. When tested for CO_2_ photoreduction, the AuPd/3DOM–TiO_2_ catalysts exhibited relatively impressive quantum yields of 0.2–0.4% at 520 nm together with excellent selectivity for the formation of methane [267].

Compared to single-component plasmonic metal structures, hybrid nanostructures have shown better optical, electronic, and magnetic properties combined with plasmonic properties [60,268,269,270,271]. This has particularly come into application in another environmental initiative: H_2_ generation. Alongside CO_2_ photoreduction, H_2_ generation is another green chemistry process. Hydrogen is the simplest element on earth and is notable for its ability to store and deliver usable energy. It has potential use for generating power in fuel cells using chemical reactions as opposed to combustion with byproducts of water and heat that are largely harmless to the environment. As a green fuel, hydrogen can be used in cars, houses, and in various other technological applications.

Many studies have found that the synergistic effect of the LSPR action of Au and the catalytic activity of Pt for H_2_ generation, can produce a strong increase in photocatalytic reaction rates. Schmuki et al. have demonstrated that single component ALD deposited Pt–TiO_2_ nanotubes show high efficiencies for H_2_ production under solar and UV light [272]. A second study, involving a slightly modified architecture of spaced TiO_2_ nanotubes in contrast to closely packed TiO_2_ nanotubes decorated with Pt nanoparticles via ALD deposition, highlights better performance efficiencies for H_2_ generation and the importance of optimized architectures for plasmonic photocatalysis [273]. Here, amounts of 171.6 µL/h (for ten ALD cycles) and 150.75 µL/h (for two cycles) are observed under UV light. Under solar light, amounts of 10.53 µL/h (for ten ALD cycles) and 9.41 µL/h (for two cycles) are observed. These results demonstrate the importance of optimized architectures for plasmonic photocatalysis with much higher photocatalytic performances observed in the spaced out nanotubes with smaller Pt nanoparticles (via lesser cycles) [273] versus the common architecture of closely packed nanotubes [272].

**Figure 23:**
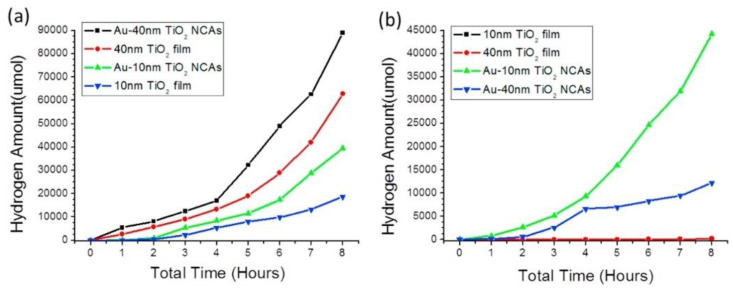
Hydrogen generation comparisons using samples with differing coating thicknesses of TiO_2_ on Au/TiO_2_ nanocrystal arrays and bare TiO_2_ thin films under (**a**) UV irradiation (Hg lamp), and (**b**) visible-light irradiation (Xe lamp) [268]. Approximating roughly by eye, under UV light, 40 nm Au TiO_2_ nanocrystal arrays generate ~90,000 µmol of hydrogen while 10 nm Au TiO_2_ nanocrystal arrays generate ~37,000 µmol of hydrogen after 8 h, respectively. Under similar conditions, the 40 nm and 10 nm bare TiO_2_ films generate ~60,000 µmol and 17,000 µmol. On the other hand, under visible radiation, the hydrogen production from the bare TiO_2_ thin films is largely negligible, while the 40 nm and 10 nm Au TiO_2_ nanocrystal arrays generate ~12,000 µmol and 45,000 µmol of hydrogen after 8 h. Essentially, compared to bare TiO_2_ films, the Au/TiO_2_ nanocrystal arrays present higher photocatalytic activity. Reprinted with permission from Ref [268] Copyright Elsevier (2016).

In order to benefit from the synergistic presence of both Pt and Au in the photocatalyst, A. Tanaka et al. [274] co-decorated nanocrystalline TiO_2_ powders with unalloyed monometallic ~13 nm sized Au NPs and ~3 nm Pt NPs using photodeposition, which stands in contrast to the previously described technique of using alloyed bimetallic nanoparticles (of which at least one is a noble metal) to form Schottky-type heterojunctions with TiO_2_ surfaces. The resulting photocatalysts displayed prominent and relatively narrow LSPR peaks centered at ca. 550 nm corresponding to the Au NPs [274]. Because the Pt NPs were clearly separate from the Au NPs instead of a mixed composition or core-shell structure, the Pt did not introduce any additional damping into the LSPR of the Au NPs. The hydrogen evolution rate closely followed the LSPR profile and exhibited a linear dependence with the light absorption. Apparent quantum efficiencies as high as 0.41% were achieved for H_2_ evolution from aqueous suspensions containing isopropanol as the sacrificial hole scavenger. While anatase TiO_2_ powders and anatase-rutile mixed phase TiO_2_ powders co-decorated with Au NPs and Pt NPs successfully evolved H_2_, pure rutile powders co-decorated with Au NPs and Pt NPs evolve negligible amounts of H_2_ [274]. This was explained on the basis of the lower CB edge of rutile TiO_2_ compared to anatase TiO_2_ which renders the hot electrons injected from Au into the rutile CB unable to reduce H^+^ to H_2_. Wu et al. [268] demonstrated hydrogen production using hexagonal close-packed core-shell Au/TiO_2_ nanocrystal arrays. Under UV and visible light irradiation, their work reported a dramatic increase in hydrogen production from 20% methanol solution achieved with the hybrid Au/TiO_2_ nanocrystal arrays in comparison with bare TiO_2_ thin films and randomly distributed Au/TiO_2_ nanocrystals (Figure 23). Wu et al. correlated the significant increase in hydrogen production to the optimal coupling of the enhanced electric field from localized surface plasmon resonances in Au/TiO_2_ nanocrystal arrays. Their results provide an effective overview of how hybrid nanostructures can be optimized for maximum LSPR enhancement to assist in photocatalytic mechanisms. A very different result was obtained by Z. Zhang et al. [275], who observed that irradiation of their TiO_2_ nanofiber–AuPt NP bimetallic plasmonic nanocomposite by 520 nm photons did not yield any hydrogen generation in spite of the same nanocomposite producing 2.33 mmol g^−1^ h^−1^ of H_2_, and outperforming bare TiO_2_ nanofibers by a factor of over 1000, under broadband illumination from a 300 W Xe lamp in an aqueous electrolyte (pH = 4) containing 0.1 M ascorbic acid as a sacrificial hole scavenger. The plasmonic nanocomposites with varying Au:Pt ratios were synthesized by electrospinning a mixture of PVP + HAuCl_4_ + H_2_PtCl_6_ + Ti(OC_4_H_9_)_4_, and exhibited strong LSPR peaks in their optical spectra centered between 540 nm and 590 nm [275]. The increase in hydrogen evolution under broadband illumination of the plasmonic nanocomposite albeit with the absence of correspondence with the LSPR profile, is strongly suggestive of improved charge separation produced by the AuPd NPs and photocatalytic activity possibly driven by an interband transition in the Au NPs.

Recent work strongly supports the use of dual particle size Au NP co-catalysts on nanostructured TiO_2_ to achieve maximum performance in photocatalytic CO_2_ reduction and H_2_ generation. Small sized Au NPs (<20 nm) strongly absorb incident photons and efficiently generate hot electron hole pairs from plasmon decay through increased Landau damping compared to radiative damping while larger nanoparticles (>50 nm) can generate higher local field enhancements at the metal–semiconductor interface [276]. Larger nanoparticles are able to sustain quadrupole and other higher order plasmon resonances that typically result in an asymmetric distribution of photogenerated charge on adjacent reaction sites [94]. The multipole plasmon-induced asymmetric charge distribution can reduce repulsion between reaction intermediates adsorbed on adjacent reaction sites and reduce the activation energy for C-C coupling reactions, which in turn, can be used to tune the selectivity of CO_2_ photoreduction toward longer chain hydrocarbon reaction products [94].

A large compendium of work exists that provide similar approaches in the use of TiO_2_–noble metal nanostructures in addressing photocatalytic reduction of CO_2_ and generation of H_2_ [205,257]. Notable efforts have motivated the modification and use of different types of architectures for TiO_2_ photocatalysts, including layered perovskites, plasmonic photocatalysts, and various morphologies of nanostructures from nanowires, nanotubes, and colloidal nanoparticle solutions [147,158,277]. While the potential of plasmonic photocatalytic systems are seen as promising, further studies with greater theoretical rigor, and a more comprehensive approach are required to develop systems with greater production rates and selectivity among those currently prevalent.

### 4.3. Photoelectrochemical Water Splitting

Photoelectrochemical water splitting has been a foundational pillar in the development of artificial photosynthetic technology. Water splitting essentially refers to the decomposition of water to molecular H_2_ and O_2_.

H_2_O → 2H_2_ + O_2_ → E^0^ = 1.23 V vs. NHE

**Figure 24:**
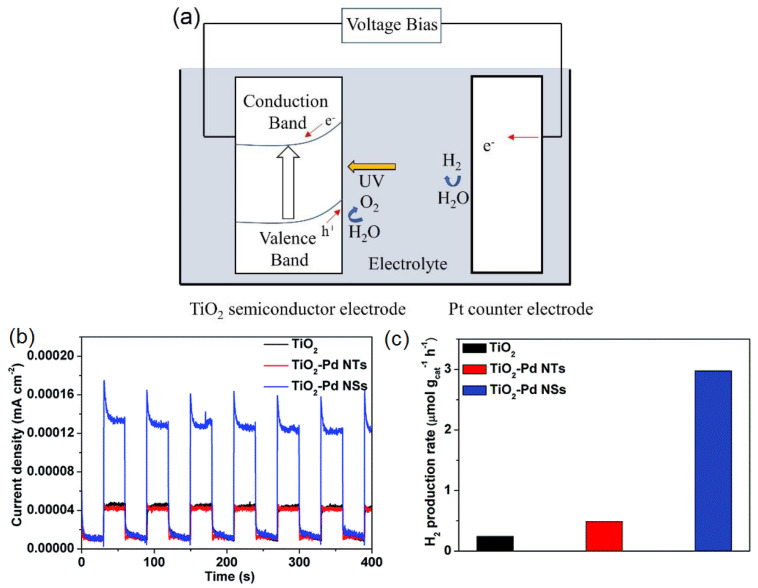
(Top, (**a**)) Schematic of the water splitting system using a wide bandgap TiO_2_ semiconductor photoelectrode and a Pt counter electrode demonstrated by Fujishima and Honda in their landmark work in 1972. Adapted with permission from Ref [50] Copyright Nature Publishing Group (1972). (Bottom, (**b**)) Photocurrent measurements made on TiO_2_–Pd nanosheet and nanotetrahedron samples and compared to that of bare TiO_2_ samples. The higher photocurrent of TiO_2_–Pd nanosheets confirms plasmonic hot electron injection, while there is not too much difference in photocurrent magnitudes between bare TiO_2_ and TiO_2_–Pd nanotetrahedrons. This provides evidence of the poor hot electron injection abilities of Pd nanotetrahedrons as compared to Pd nanosheets. (**c**) Hydrogen production rates under vis-NIR light irradiation are shown, and as can be seen the TiO_2_–Pd nanosheets exhibit photocatalytic hydrogen evolution activity unlike bare TiO_2_ and TiO_2_–Pd nanotetrahedrons [271]. These experiments provide simple insights on the application of hot electrons in photoelectrochemical water splitting. Reprinted with permission from Ref [271] Copyright Royal Society of Chemistry (2016).

In artificial systems, the above process is considered in two parts: (i) a photo- or electrochemical component that generates the oxidizing or reducing equivalents, and (ii) suitable redox catalysts that mediate the formation of the molecular gases [1]. One of the earliest works that demonstrated a complete water splitting sequence was devised by Fujishima and Honda [50]. Fujishima and Honda were the first to demonstrate that water splitting could be achieved using wide bandgap oxide n-type semiconductors that most notably include TiO_2_ or SrTiO_3_ (Figure 24). Since then, diverse solutions have emerged in achieving the same objective of water splitting through the construction of artificial systems, such as mesoporous thin films, dye-sensitized semiconductors, and more recently TiO_2_–noble metal nanostructure composites [57,244,278].

Noble metal nanorods (NRs) offer two major advantages for hot electron-driven plasmonic water-splitting—(i) longer wavelength longitudinal dipole resonances enabling the harvesting of red and near-infrared photons in the service of water photolysis and (ii) spectral separation of the interband transition from the dominant LSPR peak. Consequently, a number of reports have attempted to fabricate Au NR–TiO_2_ heterojunction to exploit these advantages. A significant advance in this sub-area was made by Y. Nishijima et al. [279], who obtained external quantum efficiencies of up to 8% (λ_max, EQE_ ~ 1000 nm) and internal quantum yields (normalized for photon absorption) of up to 15% for photocurrent generation in aqueous electrolytes using a periodic array of 240 nm × 100 nm × 40 nm Au NRs patterned on to the surface of a rutile single crystal wafer using electron beam lithography and vacuum deposition followed by liftoff. The combination of contact with the high permittivity TiO_2_ substrate coupled with collective modes produced a redshift of the fundamental transverse mode LSPR to ca. 650 nm, while the dominant longitudinal mode showed up in the extinction spectrum at ca. 1000 nm. Impressive as these quantum yield values are for long wavelength photons, EQE values of 20% and IQE values of 60% were obtained for 450 nm photons which can only excite interband transitions in gold since neither surface plasmons nor rutile TiO_2_ significantly absorb at these wavelengths. The IPCE closely followed the LSPR extinction profile up to a wavelength of 1300 nm, indicating that even 0.9 eV photons were able to successfully generate hot electron-driven photocurrents in an aqueous electrolyte containing 1.2-benzenediol as the hole scavenger [279].

**Figure 25:**
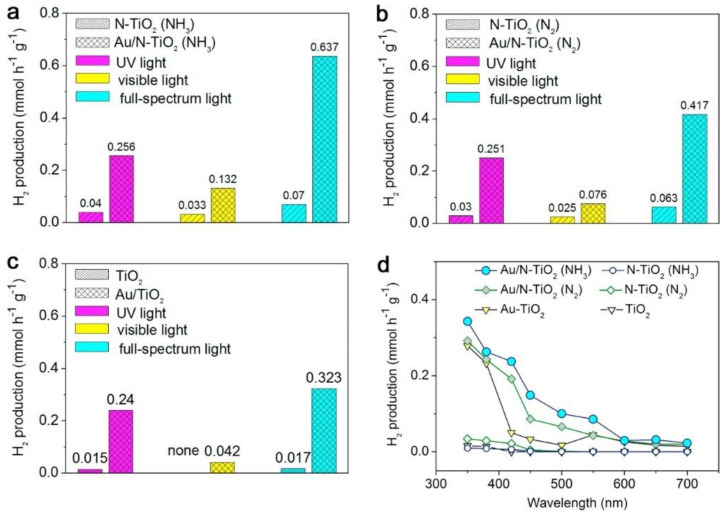
Photocatalytic hydrogen evolution, as presented by Wang et al. [280] for bare and composite bowl nanoarray and nanoparticle structures including TiO_2_, N-doped TiO_2_, Au-nanoparticles-confined n-doped TiO_2_ under different irradiation conditions (UV, Visible, and full-spectrum light): (**a**) for structures annealed in NH_3_, (**b**) for structures annealed in N_2_, and (**c**) for un-doped structures. (**d**) In all cases the plasmonic composite bowl nanoarray structures provide higher H_2_ production rates as opposed to the bare counterparts in TiO_2_, and N-doped TiO_2_ photocatalysts. Reprinted with permission from Ref [280] Copyright Elsevier (2016).

Wang et al. presented work on a facile surfactant-free nanofabrication technique involving dense Au nanoparticles in *n*-doped TiO_2_ bowl nanoarrays [280]. The integration of plasmonics in the system is quite obvious, as by tweaking the TiO_2_ bandgap to overlap the plasmonic band of Au nanoparticles, they are able to ensure the plasmonic resonant energy charge transfer processes complement well with the Schottky junction to produce enhanced photocatalytic water splitting. This is further evidenced by the high H_2_ production rates reported by the group with 637 µmol/gh under full spectrum analysis, and 132 µmol/gh for the visible spectrum (Figure 25). Once more, the rational use of hybrid structures for photocatalysis is motivated in this article. In a similar fashion, Zhang et al. demonstrated the use of hybrid structures for photoelectrochemical water splitting in their assembly of plasmonic Au nanocrystals coupled with bottom-up fabricated TiO_2_ nanotube photonic crystals on TiO_2_ nanotube photoelectrodes and achieved a maximum quantum efficiency of 8% at 556 nm [281]. Here too there is a motivation towards the matching of the LSPR resonance to the photonic band gap of the photonic crystal, such as to enhance the LSPR intensity of the Au, boosting hot electron injection from the Au nanocrystals into the TiO_2_ conduction bands, and thus leading the way to enhanced water splitting performance under visible light. Under visible light irradiation, their design is shown to produce a photocurrent density of ~150 µA/cm^2^, which is considered the highest value to be reported in any plasmonic Au/TiO_2_ system under visible light illumination.

Rather than the hot electron equilibration processes discussed in Section 2.2, it is the transfer of holes from the plasmonic nanoparticles to electrolyte ions in practical photoelectrochemical devices which is a major source of carrier losses. When 50 nm Au NPs were formed by photodeposition into nanocrystalline TiO_2_ with an anatase particle size of ~20 nm, the resulting TiO_2_–Au heterojunctions exhibited a maximum quantum yield of ~1% when deployed in a photoelectrochemical cell containing I^−^ ions that functioned as hole acceptors [282]. The reported quantum yield for charge separation for the same photocatalyst improved to a remarkable 26% at 550 nm [87], by using a N_2_- acetonitrile and ethylene glycol (*v*/*v*: 60/40) solution containing 0.1 M LiNO_3_, 0.1 M FeCl_2_, and 0.05 M FeCl_3_ (redox couple), to which 0.2 M nitrobenzoic acid was added in order to passivate surface traps in TiO_2_. These studies clearly demonstrate the importance of hole extraction in hot electron based plasmonic photoelectrochemical cells.

The potential of plasmon-enhanced photocatalytic systems for diverse environmentally significant applications, as discussed in these representative examples, is obvious. At the crux of all plasmonic photocatalytic systems is the generation of hot electrons or holes and their successful injection into a semiconductor photocatalyst. Still, there is much to be understood on the origin of the mechanism that drives these hot charge carriers toward their use in photocatalytic reactions. The following section motivates this discussion by reconciling the mystery behind two major mechanisms that have been identified as the origin of injection efficiencies in TiO_2_–noble metal nanostructured heterojunctions.

## 5. Mystery of the Action Spectrum: Reconciling Interband Transitions with Localized Surface Plasmon Resonances

**Figure 26:**
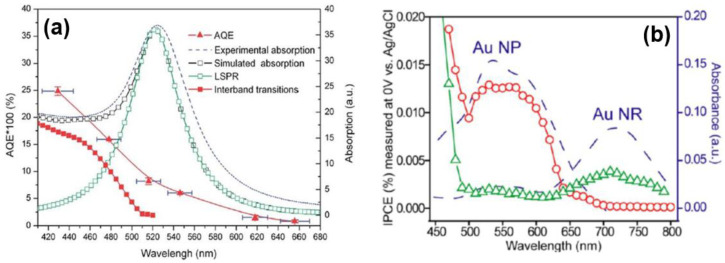
(**a**) A study of the apparent quantum efficiencies, by Liu et al. [158], to determine the driving force in photocatalysis of O_2_ evolution on 1.1% Au/SrTiO_3_ at various wavelengths of light. The important observation from these results being that water oxidation over the Au/SrTiO_3_ composite is not driven primarily by LSPR related phenomena. This is noted by the fact that the AQE curve for O_2_ evolution bears a strong resemblance to that of interband transitions suggesting that the visible light photosensitization effect of Au arises mainly from interband transitions. This fact is further supported in the results by the substantially negligible O_2_ evolution over Ag/SrTiO_3_ under visible light [158] as interband transitions of Ag are excited only in the UV spectrum through plasmon resonance, and observed in the visible region. Reprinted with permission from Ref [158] Copyright Royal Society of Chemistry (2014). (**b**) Shows the quantum efficiencies at a bias of 0 V vs Ag/AgCl for photoelectrochemical water splitting using plasmonic gold nanoparticle decorated TiO_2_ nanowires and gold nanorod decorated TiO_2_ nanowires, which follow the LSPR absorption profile. Reprinted with permission from Ref [286] Copyright American Chemical Society (2013).

**Figure 27:**
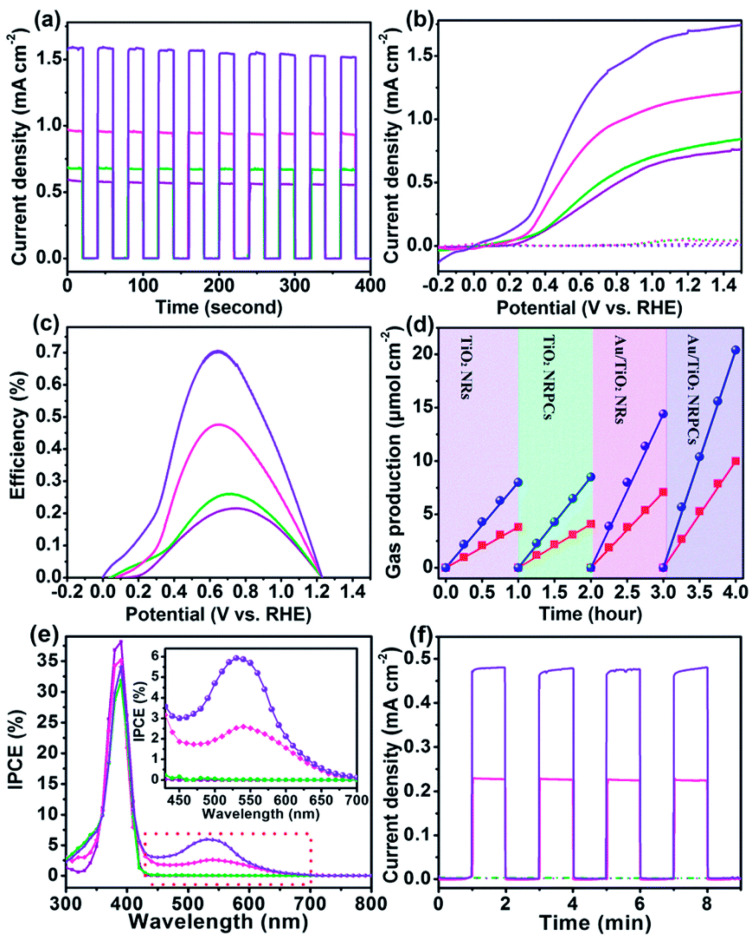
Comparing the PEC properties of four different photoanodes: TiO_2_ nanorods (dark purple solid line), TiO_2_ nanorod photonic crystals (green solid line), Au/TiO_2_ nanorods (pink solid line), and Au/TiO_2_ nanorod photonic crystals (pale purple solid line) [257]. (**a**) Chronoamperometry measurements performed at an external potential of 1 V vs. Reversible Hydrogen Electrode (RHE). The on/off circles indicate simulated sunlight illumination. (**b**) Linear sweep voltammetry measurements for a scan rate of 50 mV/s under dark (dotted lines) and illuminated conditions. (**c**) Photoconversion efficiency as a function of applied potential versus RHE. (**d**) The resultant evolution of H_2_ and O_2_ gases for the four photoanodes under simulated sunlight illumination. Of particular note are the IPCE results in (**e**) which displays the shapes of the IPCE active spectra of the various Au/TiO_2_ composite photoanodes. PEC activity within the visible light regime is observed due to the presence of the Au noble metal nanoparticles. Furthermore, the shapes of the IPCE spectra as shown in the inset are noted to be similar to that of LSPR absorption spectra of Au nanoparticles. (**f**) Amperometric I-t curves for the four different photoanodes for repeated on-off cycles of simulated sunlight. Reprinted with permission from Ref [257] Copyright Royal Society of Chemistry (2014).

Various experimental results and figures have been presented over the course of this review on the applications of hot electrons in plasmonic photocatalysis. At the fundamental level, the biggest issue for plasmonic photocatalysis involves the low quantum efficiencies evidenced in hot electron injection into the *n*-type semiconductor [123,258,283,284]. The incident photon-to-electron conversion efficiency is a metric that evaluates the quantum efficiencies of plasmonic photocatalytic systems. The IPCE is the ratio of the number of collected photoelectrons that perform useful work (in this case drive desirable a chemical reaction) to the number of incident photons. In hot electron photoelectrochemical cells, the photocurrent is typically a good measure of the final number of electrons performing useful work (the assumption here is that of a high Faradaic efficiency). Therefore, the IPCE is usually presented as a percentile figure evaluated by the formula IPCE [%] = 1.24 × 10^5^ [I_1_/(P · λ)], where I_1_ (A) is the photocurrent, P (W) is the incident light power, and λ (nm) is the wavelength of the incident light (Figure 26 and Figure 27) represent IPCE graphs in two experimental cases, one involving tests that identified the active photoelectrochemical spectra for various TiO_2_ and Au/TiO_2_ photoanodes [257], while the other provides results on the O_2_ evolution over 1.1% Au/SrTiO_3_ at different light wavelengths [158]. A unique question that arises from these results as well as others prevalent in the literature on plasmonic photocatalysis, is the origin of the mechanism which essentially drove the electrons represented in the action spectrum toward photocatalytic reaction. Two major responses have been identified to correspond with injection efficiencies for visible light sensitization in nanostructured Au–TiO_2_ heterojunctions: interband transitions of valence electrons to an available conduction band above the Fermi level (Figure 26) or the excitation of valence electrons to a collective oscillatory state around the Fermi level, otherwise known as the localized surface plasmon resonance. (Figure 27) This begs the question why the plasmonic enhancement phenomenon seems to primarily follow, in some cases, the profile of interband transitions of *d*-band electrons within the noble metal nanoparticle [285], and in other cases, that of the localized surface plasmon resonance.

A definite answer to this fundamentally important observation has so far been elusive, though a starting hypothesis revolves around the nature and characteristic properties of the nanoparticle being utilized. It has been shown in recent studies that the energy profile of plasmon-induced hot electrons is sensitive to the composition of the particle [65,66,217,287,288]. These studies present that intraband excitations (transitions within the conduction band) induced by visible light produce hot electrons and holes, while interband excitations (from the *d*-band of the metal to states above the Fermi level) produce high-energy holes but low energy electrons. This is particularly evident when one compares plasmonic systems involving Au and Ag. Due to the higher interband energy of Ag nanoparticles, intraband transitions are favored, while the lower interband energy in Au results in favoring hot electrons produced via interband transitions.

Apart from the electronic structure of the metal nanoparticle, numerous other properties, including the loading amount and size [151], architecture of the noble metal nanoparticle [58,60,181,238], the amplitude of UV irradiation [151], the structural distribution of the noble metal nanoparticle forming the semiconductor heterojunction (composite plasmonic nanostructure systems where the plasmonic noble metal is embedded within the semiconductor nanostructure as opposed to decorating the nanostructure have resulted in greater performance efficiencies), all influence the mechanism of hot electron injection in noble metal nanoparticle–semiconductor heterojunctions [195,249,289].

Ratchford et al. [130] emphasize the estimation of four factors that are integral to theoretical studies tackling the difficult problem of calculating the charge injection efficiency at a noble metal nanoparticle–semiconductor heterojunction: the excited charge carrier energy distribution, the probability that the excited charge carriers reach the interface, the number of excited charge carriers that have sufficient energy and momentum to cross the interface barrier, and the transmission probability across the interface for the charge carriers with sufficient momentum [130]. Using a combination of transient absorption spectroscopy and mid-IR transient absorption spectroscopy Ratchford et al. show that for Au nanoparticles fully embedded within TiO_2_ (as opposed to Au NPs decorating TiO_2_), injection efficiencies in the 25–45% ranges are achievable. Additionally, they are able to provide for comparisons with an upper-limit estimate of the electron injection efficiency showing the significance of yet another important property of charge carrier transport in an interplay with prior listed properties, in momentum matching conditions at the nanoparticle–semiconductor interface.

Charge carrier momentum is a crucial factor in transport efficiency. Transport efficiency is the probability that the charge carrier reaches the interface and is itself dependent on where the charge carrier is generated in the nanoparticle and its mean free path, which is a function of the particle’s energy. After reaching the interface, in addition to having sufficient energy to overcome the Schottky barrier, electrons must also have the correct momentum to cross the interface and be transmitted rather than reflected. These conditions are codependent on the nanoparticle geometry and size. As Ratchford et al. [130] point out, in the case of Au nanoparticles embedded in TiO_2_ (a system realized by Farsinezhad et al. [195]), wherein the nanoparticle dimensions are less than the mean free path of electron–electron scattering most of the excited electrons are allowed to reach the interface. Similarly, the fact that the nanoparticles are fully embedded within the semiconductor provides greater opportunities for the excited electron to be injected into the semiconductor, and last but not least, the small size of the nanoparticles assists in relaxing momentum conservation requirements at the interface. In layman terms, a simple picture can be derived from the established statements. Charge carriers not only require sufficient energy but also the right momentum to cross the Schottky barrier and deposit onto the semiconductor. In the case of metal nanoparticles decorating a semiconductor, only a portion of electrons would have their momentum in the right direction, namely, to cross the interface. Meanwhile, if the metal nanoparticles are embedded in the semiconductor, the momentum distribution is constrained to be isotropic by way of the architecture. In such a configuration the momentum conservation condition is relaxed, as there is an interface everywhere around the metal nanoparticle, and the dependency on direction is lost as no matter what direction the electrons travel, there will always be an interface, and the surrounding semiconductor, thus allowing for greater injection efficiencies. There are also indications that formation of a heteroepitaxial interface between Au and TiO_2_ [290] or the use of a molecular linker to covalently bond Au NPs to TiO_2_ [291] might be more effective in increasing hot carrier lifetimes than creating disordered Au–TiO_2_ junctions in direct contact, although this is not yet conclusive.

One such work that incorporates this knowledge is presented in the work of Naldoni et al. [292] showing photoelectrochemical water oxidation on brookite TiO_2_ nanorods. Naldoni et al. particularly emphasize how the location of Au nanoparticle deposition on the TiO_2_ nanorods enhances the photoelectrochemical water oxidation process. Using a combination of electrochemical and ultrafast optical spectroscopy, electron-hole recombination phenomena are discovered to be at the core of the water oxidation activity. Furthermore, they show that a preferential distribution of Au nanoparticles decorating the electrode/wafer interface results in a higher photocurrent as opposed to when the Au nanoparticles are distributed along the film thickness. This is observed as the Au surface decoration provides for 4 orders of magnitude increase in hot electron lifetime (up to ns) due to efficient site hopping on brookite lateral facets, thus strengthening the plasmon-enhanced solar water oxidation process while simultaneously averting common damping processes that reduce hot charge carrier lifetimes (usually in the ps and fs range).

Interband transitions can be directly excited by incident photons of sufficient energy. These transitions can also occur due to the non-radiative decay of particle plasmons into electron-hole pairs through interband excitations (involving the *d* band in Au for instance) which require a minimum energy of 1.8 eV in Au [124]. Thus, the resonance frequency corresponding to the LSPR peak is important in determining whether the plasmons can decay through interband transitions or not. In anisotropic nanoparticles, such as high aspect ratio colloidal gold nanorods, the longitudinal plasmon resonance occurs at energies of 1.2–1.7 eV [293], thus precluding the possibility of interband non-radiative plasmon decay. Likewise, the interband decay mechanism of particle plasmons is also precluded for gold spheres embedded in a high refractive index medium which exhibit LSPR peaks that are highly red-shifted from the wavelength maximum corresponding to surface plasmon resonance in an air medium (~530 nm). Therefore, for plasmonic nano-architectures, the shape of the noble metal nanoparticles and the permittivity of the surrounding medium are therefore also likely to play a role in determining whether the external quantum yields for plasmonic photocatalysis follow the LSPR profile or the profile of interband transitions in gold.

Nevertheless, these discussions do not provide a complete answer to our question on the selectivity and competition of charge carrier injection mechanisms between LSPR promoted transitions and interband transitions in plasmonic composite systems. Thus, it is critical that future experiments also consider the various facets of charge transfer, interfacial charge carrier injection mechanisms, and their prominent role in the design of plasmonic hot electron devices [152,161,234,294,295,296].

## 6. Conclusions

This review provides a survey of the current landscape of research in the field of plasmonic photocatalysis with specific focus on the development of artificial photosynthetic systems. The competing mechanisms that underpin hot electron phenomena have been discussed at length, and various advanced techniques to characterize hot electrons have been introduced and explained. Deriving from their ability to take advantage of highly energetic charge carriers (hot electrons), metal/semiconductor hybrid photocatalytic systems have demonstrated great potential in addressing the issues involving inefficient photocatalytic performance of certain semiconductors, and the failure to effectively utilize the full extent of the solar spectrum. TiO_2_–noble metal nanoparticle heterojunctions have shown encouraging results in implementing said objectives, albeit with consistent growth in research that have identified and paved the way for suitable modifications and enhancements through diverse structures and material considerations. The quantum yields achieved for LSPR-driven photocatalytic reactions by a number of experimental reports, and the estimates of quantum efficiencies from spectroscopic studies for hot electron injection from noble metal nanoparticles into the conduction band of TiO_2_, significantly exceed the theoretical limits placed by the conventional sequential mechanism of surface plasmon dephasing and hot electron equilibration. The theory for alternative approaches, such as chemical interface damping and plasmon-enhanced interfacial charge transfer is not well-developed and presents opportunities for further research. A major contribution of our review has been to highlight the discrepancy between plasmonic photocatalytic systems often constituted of similar or identical materials wherein the quantum yield follows the profile corresponding to interband transitions in some cases and follows the LSPR profile in other cases. We have attempted to explain this discrepancy by pointing out the importance of momentum conservation in addition to energy conservation in nanostructured noble metal–semiconductor heterojunctions. We presented the hypothesis that photocatalytic systems wherein the noble metal nanoparticles are partially or fully embedded or within the semiconductor or else sandwiched between two semiconductor surfaces, are more likely to achieve efficient hot electron injection yields with a photoresponse that follows the LSPR profile compared to systems where the noble metal nanoparticles merely decorate the surface of the semiconductor which are more likely to exhibit action spectra that follow the profile of interband transitions.

Looking toward the future, there is a greater challenge that must be tackled in gaining a fundamental understanding of the charge transfer mechanisms involved in hot carrier photocatalytic systems. This is with reference to the lack of knowledge that surrounds our comprehension of the microscopic origin of the plasmon-induced hot carrier formation on optically excited plasmonic nanoparticles, and the subsequent charge dynamics that come into play; examples include the yield of hot carrier formation due to the decay of particle plasmons in different system, the role of adsorbates and the conditions required (apart from the presence of a Schottky barrier) to achieve a high efficiency of injection of hot electrons into the conduction band of TiO_2_. These gaps in knowledge provide the motivation for further fundamental studies on the subject as only by sufficiently probing the hot electron effect can the stage be set for the optimization of current and future artificial photosynthetic systems that capitalize on the advantages that plasmonic noble metal–semiconductor composite systems offer.
